# Costs and Scale-Up Costs of Integrating HIV Self-Testing Into Civil Society Organisation-Led Programmes for Key Populations in Côte d'Ivoire, Senegal, and Mali

**DOI:** 10.3389/fpubh.2021.653612

**Published:** 2021-05-24

**Authors:** Marc d'Elbée, Métogara Mohamed Traore, Kéba Badiane, Anthony Vautier, Arlette Simo Fotso, Odé Kanku Kabemba, Nicolas Rouveau, Peter Godfrey-Faussett, Mathieu Maheu-Giroux, Marie-Claude Boily, Graham Francis Medley, Joseph Larmarange, Fern Terris-Prestholt

**Affiliations:** ^1^Department of Global Health and Development, Faculty of Public Health and Policy, London School of Hygiene and Tropical Medicine, London, United Kingdom; ^2^Solthis, Abidjan, Côte d'Ivoire; ^3^Solthis, Dakar, Sénégal; ^4^Centre Population et Développement (Ceped UMR 196), Institut de Recherche pour le Développement (IRD), Université de Paris, Inserm (ERL 1244), Paris, France; ^5^Solthis, Bamako, Mali; ^6^Joint United Nations Programme on HIV/AIDS (UNAIDS), Geneva, Switzerland; ^7^Clinical Research Department, Faculty of Infectious and Tropical Diseases, London School of Hygiene and Tropical Medicine, London, United Kingdom; ^8^Department of Epidemiology, Biostatistics, and Occupational Health, School of Population and Global Health, McGill University, Montréal, QC, Canada; ^9^Department of Infectious Disease Epidemiology, Medical Research Council Centre for Global Infectious Disease Analysis, Imperial College London, London, United Kingdom

**Keywords:** costs and cost analysis, scale-up, HIV self-testing, key populations, knowledge of HIV status, diagnosis, screening, West Africa

## Abstract

Despite significant progress on the proportion of individuals who know their HIV status in 2020, Côte d'Ivoire (76%), Senegal (78%), and Mali (48%) remain far below, and key populations (KP) including female sex workers (FSW), men who have sex with men (MSM), and people who use drugs (PWUD) are the most vulnerable groups with a HIV prevalence at 5–30%. HIV self-testing (HIVST), a process where a person collects his/her own specimen, performs a test, and interprets the result, was introduced in 2019 as a new testing modality through the ATLAS project coordinated by the international partner organisation Solthis (IPO). We estimate the costs of implementing HIVST through 23 civil society organisations (CSO)-led models for KP in Côte d'Ivoire (*N* = 7), Senegal (*N* = 11), and Mali (*N* = 5). We modelled costs for programme transition (2021) and early scale-up (2022–2023). Between July 2019 and September 2020, a total of 51,028, 14,472, and 34,353 HIVST kits were distributed in Côte d'Ivoire, Senegal, and Mali, respectively. Across countries, 64–80% of HIVST kits were distributed to FSW, 20–31% to MSM, and 5–8% to PWUD. Average costs per HIVST kit distributed were $15 for FSW (Côte d'Ivoire: $13, Senegal: $17, Mali: $16), $23 for MSM (Côte d'Ivoire: $15, Senegal: $27, Mali: $28), and $80 for PWUD (Côte d'Ivoire: $16, Senegal: $144), driven by personnel costs (47–78% of total costs), and HIVST kits costs (2–20%). Average costs at scale-up were $11 for FSW (Côte d'Ivoire: $9, Senegal: $13, Mali: $10), $16 for MSM (Côte d'Ivoire: $9, Senegal: $23, Mali: $17), and $32 for PWUD (Côte d'Ivoire: $14, Senegal: $50). Cost reductions were mainly explained by the spreading of IPO costs over higher HIVST distribution volumes and progressive IPO withdrawal at scale-up. In all countries, CSO-led HIVST kit provision to KP showed relatively high costs during the study period related to the progressive integration of the programme to CSO activities and contextual challenges (COVID-19 pandemic, country safety concerns). In transition to scale-up and integration of the HIVST programme into CSO activities, this model shows large potential for substantial economies of scale. Further research will assess the overall cost-effectiveness of this model.

## Introduction

In Western and Central Africa, 5 million people are living with HIV, representing a prevalence of 1.4% in 2019 (1). As in most countries of the region, the epidemic is mixed in Côte d'Ivoire, Senegal, and Mali, with national prevalence in 2018 ranging between 0.4 and 2.6% and much higher prevalence at 5–30% in hard-to-reach key populations (KP) including female sex workers (FSW), men who have sex with men (MSM), and people who use drugs (PWUD) (1). In 2019 in Western and Central Africa, HIV prevalence was 10% for FSW, 14% for MSM, and 5% for PWUD (1). Because of the HIV prevention gap among these groups, KP contribute mostly to HIV transmission (2–4).

UNAIDS has set targets for 95% of people living with HIV to know their status, 95% of known HIV-positive individuals to be on antiretroviral therapy (ART), and 95% of those on ART to have their viral load suppressed by 2030 (5). Despite significant progress on the proportion of individuals who know their HIV status (increase from 4% in 2000 to 67% in 2020), Western Africa remains far below the first 90 UNAIDS target, with disparities observed between Côte d'Ivoire (76%), Senegal (78%), and Mali (48%) in 2020 (6).

Conventional facility-based HIV testing services (HTS) does not adequately reach those KP due to stigma, discrimination, and health services not responding to needs specific to each group. Local civil society organisations (CSO) providing mostly community-based HIV testing services using peer educators have proven successful in reaching the core members of these populations, linking, and retaining them into care (7, 8).

HIV self-testing (HIVST) is defined as a process where a person collects his/her own specimen (oral fluid or blood), performs an HIV test and interprets the result, often in private (9). Following promising demonstration projects in Eastern and Southern Africa (10–15), HIVST was introduced in 2019 as a new testing modality in West Africa with the ATLAS project (*Auto Test VIH, Libre d'Accéder à la connaissance de son Statut*) (16). The project is led by the French non-governmental organisation Solthis—namely international partner organisation (IPO) in this study—in consortium with the Institut de Recherche pour le Développement, Ministries of Health, and local implementing CSO in Côte d'Ivoire, Senegal, and Mali. HIVST has the potential to overcome some of the existing structural barriers to testing and to increase diagnosis coverage among KP (primary distribution) and their peers, sexual partners and clients (secondary distribution) not reached by conventional HTS (17, 18).

OraQuick® HIV self-tests have been subsidised by the Bill and Melinda Gates Foundation, then proposed by Orasure Inc. at US$2 per kit in 50 low- and middle-income countries for public sector distribution (19). However, HIVST is still around twice the price of standard HIV rapid diagnostic tests currently used for HIV testing in Africa. In southern Africa, HIVST increased diagnosis coverage and showed potential value for money for key populations as a complement to current testing approaches (9, 10, 20).

In this study, we estimate the costs of implementing HIVST through CSO for KP in Côte d'Ivoire, Senegal, and Mali. We also assess the costs of scaling up this model to guide project national scale-up, propose costed operational plans, and inform on the sustainability of this distribution model.

## Materials and Methods

### Intervention Setting

**Figure 1:**
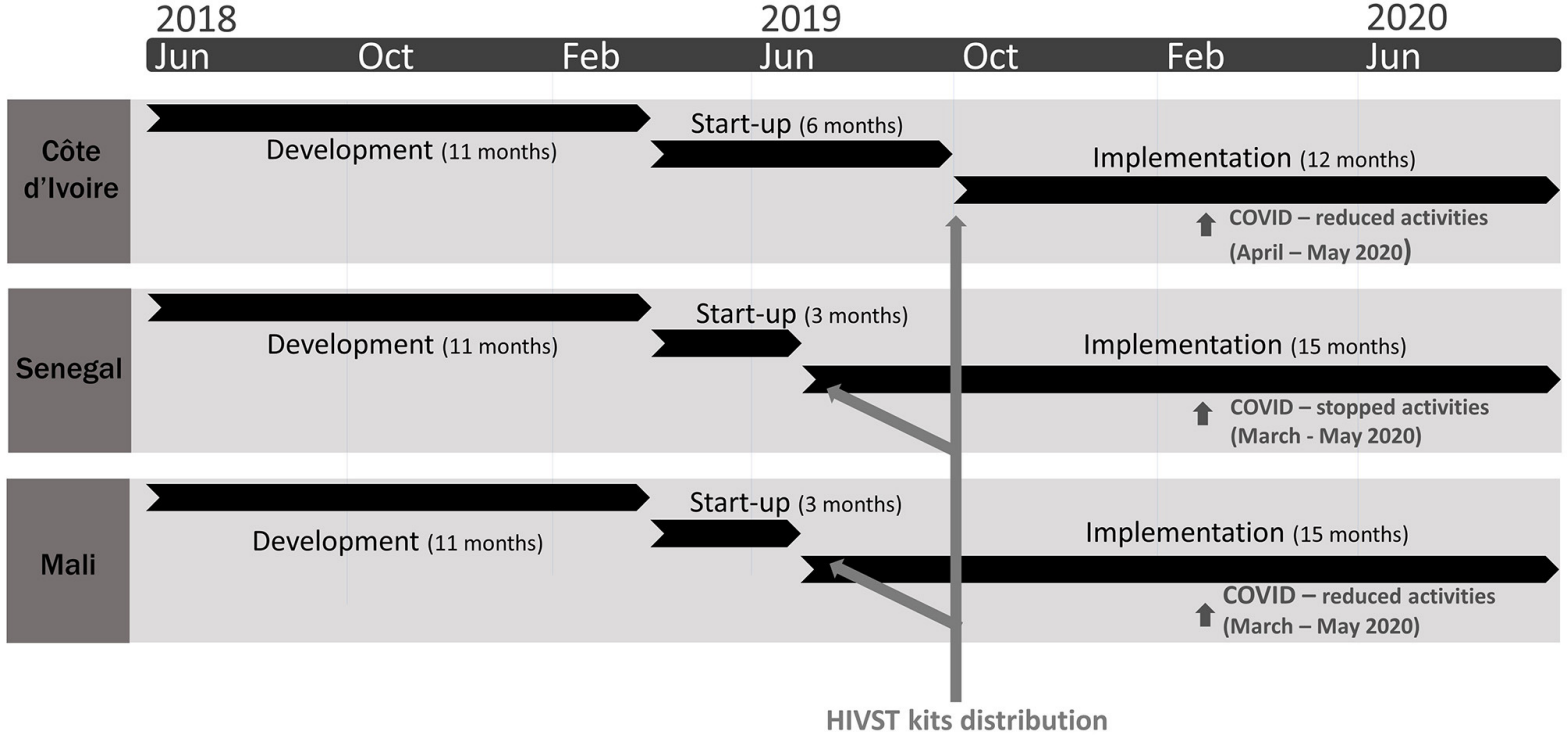
Description of the ATLAS project's three HIV self-testing (HIVST) deployment phases in Côte d'Ivoire, Senegal, and Mali over 2018–2020.

HIVST kits were distributed through 23 CSO across Côte d'Ivoire (*N* = 7), Senegal (*N* = 11), and Mali (*N* = 5) from July 2019 to September 2020. Implementing partners' key characteristics are presented in Table 1. The deployment strategy identified three sequential intervention phases: (1) *development phase (June 2018–March 2019)*: all activities that identify sustainable distribution models for each country, to fully integrate HIVST into existing programmes; (2) *start-up phase [April 2019–July 2019 (Senegal/Mali), - October 2019 (Côte d'Ivoire)]*: adaptation of self-testing information materials to the local context, development of training manuals, training of HIVST providers, sensitisation of key actors and building partnerships with local partners (regardless of when the costs were incurred), and other start-up costs; and 3) *early implementation phase (up to September 2020)*: demand creation, HIVST kits distribution, and project supervision (Figure 1). In each country, all CSO did not start HIVST kits distribution at the same time, and this was accounted for in the cost analysis by adjusting the length of the implementation period by distribution channel. We costed community-based activities used by CSO for reaching KP and excluded facility-based costs corresponding to HIVST kits provision through index testing and sexual health consultations, accounting for a small proportion of CSO activities and outside the scope of this analysis. CSO1 (Senegal) is not technically a CSO but a public facility included in the analysis because they provide community-based services to PWUD.

**Table 1:** Overview of 1the ATLAS project's implementing partners in Côte d'Ivoire, Senegal, and Mali.

**Country**	**Administrative**	**Number of districts**	**Civil society**	**Distribution**	**Number of trained**	**HIVST kits**
	**region**	**covered**	**organisation**	**channel**	**HIVST providers**	**HIVST providers**
Côte d'Ivoire	Gbôklé, Nawa, San-Pédro	2	CSO1	FSW	13	9,605
				MSM	4	4,172
	Abidjan 1	2	CSO2	FSW	29	9,175
	Abidjan 2	2	CSO3	FSW	20	15,944
				MSM	6	6,812
				PWUD	9	4,230
	Mé, Abidjan 1	2	CSO4	MSM	7	2,177
	Sud Comoé	1	CSO5	FSW	6	2,261
				MSM	5	1,370
	Mé, Sud Comoé	2	CSO6	FSW	13	5,181
				MSM	8	2,511
	Gbôklé, Nawa, San-Pédro	2	CSO7	FSW	8	7,044
				MSM	3	4,406
**Sub-total**					**131**	**74,888**
Senegal	Dakar, Thiès	11	CSO1	PWUD	22	1,862
	Dakar, Thiès, Ziguinchor	18	CSO-Associations	FSW	25	1,540
				MSM	33	2,933
	Dakar, Thiès	9	CSO-mobile clinics	FSW	4	810
	Dakar, Thiès, Ziguinchor	17	CSO-independent distributors	FSW	16	4,320
				MSM	12	2,400
				PWUD	4	160
**Sub-total**					**116**	**14,025**
Mali	Bamako, Sikasso, Koulikoro, Kayes, Segou	7	CSO1	FSW	15	11,250
				MSM	14	4,813
	Bamako, Segou, Sikasso, Kayes, Koulikoro	11	CSO2	FSW	78	22,400
				MSM	20	3,360
	Bamako, Segou, Sikasso	5	CSO3	FSW	31	20,910
	Kayes, Koulikoro	12	CSO4	MSM	19	12,321
	Sikasso	2	CSO5	FSW	7	4,623
				MSM	7	2,139
**Sub-total**					**191**	**81,816**
TOTAL					438	170,729

*HIVST, HIV Self-Testing kit; FSW, Female Sex workers; MSM, Men who have Sex with Men; PWUD, People who use drugs*.

### Cost Data Collection and Analysis

The costing teams followed the Global Health Cost Consortium guidelines and collaboratively analysed data, ensuring consistency of methods across countries (21–23). We used the provider's perspective. We conducted an incremental cost analysis, where only additional resources needed to introduce HIVST to existing service provision were considered. These incremental costs were collated from the IPO and implementing partners' financial expenditures and each line item was categorised by input type and distribution model (top-down costing approach) (24). Inputs were categorised into start-up, capital, and recurrent costs. Inputs were allocated to distribution sites following predefined allocation factors, based on project monitoring and evaluation data, including the percentage of HIVST distributors in each site, estimated cohort size of HIV-positive patients followed by the CSO, percentage of kits distributed, and percentage of direct expenditures, which is a weighted average of the preceding allocation factors. Further details on the methods and allocation factors can be found in Appendix Table 1, and elsewhere (25–27). To estimate economic costs, the expenditure analysis was complemented by a valuation, with market prices or financial data provided by the implementers, of all other resources used in the delivery model (donated services such as personnel time at the CSO headquarters and in the field, not paid by the ATLAS project). Finally, a time-motion study was conducted to observe staff providing HIVST alongside other services and allocate personnel costs based on the time spent on each activity (28, 29). The HIVST kit cost was US$2.68 for Côte d'Ivoire and US$3.08 for Senegal and Mali. Start-up, training, and all other capital costs were annualised using a discount rate of 3%. All costs were estimated in 2020 USD dollars using annual exchange rates. Total costs and average cost per kit distributed were estimated at the country level, at the CSO level and per channel.

### Sensitivity Analysis of Costs

We conducted a series of one-way sensitivity analyses, using tornado diagrams, to assess the impact of key cost assumptions on the average cost per HIVST kit distributed. We varied the discount rate used to annualised costs to 0 and 16% (base case is 3%) to capture the impact of not discounting or using a higher local central bank discount rate such as in Mali (30). We evaluated the impact of applying alternative allocation factors that is swapping percentage trained distributors to percentage cohort size for IPO expenditures. We varied annualisation (economic life years) time frames: training & sensitisation were varied between 1 and 3 years (base: 2 years), project development life between 5 and 15 years (base: 10 years), and start-up life (training, sensitisation and other costs incurred during this phase) between 2.5 and 7.5 years (base: 5 years) to assess the impact of the assumed project life years on costs. For Senegal only due to data availability, we swapped the allocation of field-based personnel costs from using percentage HIVST time observed during the time-motion study to using percentage HIVST time reported by study participants. Finally, episodes of violence against MSM occurred during the study period, and CSO had to suspend their activities in Senegal and Mali. The COVID-19 pandemic also led to reduced/suspended activities (Figure 1), therefore we also estimated the average cost per target HIVST distribution volumes.

### Scale-Up Cost Model and Scenario Analysis

We also modelled costs at scale-up when HIVST kit distribution volumes would increase following each country's National Strategic Plan for HIV testing to predict the variation of average cost between the implementation and scale-up phases. The production function, developed by Cobb and Douglas, describes the relationship between outputs and factors of productions (inputs) (31). Accounting cost functions follow step-by-step the intervention production process as close as possible to reality (22, 32). They identify fixed and variable costs, typically assumed to vary linearly with the scale such as that used in input-output analysis as originally developed by Leontief (33, 34). It should be noted that with the exception of training costs (variable cost) and sensitisation costs (fixed cost) considered in the scale-up model, all other costs incurred during the development and start-up phases are considered one-off costs incurred at the start of the programme and therefore, are excluded from the costs of scaling-up. The model algebra is presented here, the detailed model structure listing fixed and variable costs is presented in Table 2.

$$\begin{array}{l} C=\sum_{j}\left(FC_{j}+VC_{j}\right)\\ \text{with }VC_{j}=UC_{j}\cdot S_{j} \end{array}$$

**Table 2:** Model structure—Accounting cost function.

**Intervention level**	**Type of costs**	**Cost inputs**	Scale variable^*^
International	Fixed costs	S2. Sensitisation—Coordination R1. Personnel and Per diems—Headquarters IPO coordination	
	Variable costs	None	
National	Fixed costs	C1. Buildings and storage C2. Equipment C3. Vehicles C4. Other capital costs S2. Sensitisation—IPO country R2. Personnel and Per diems—Headquarters IPO country	
	Variable costs	S1. Trainings (start-up phase only)	Number of *new* providers to train
		R6. Vehicle operation and maintenance/transportation	Total number of HIVST providers
		R7. Building operation and maintenance	Total number of HIVST providers
		R8. Other recurrent costs	Total number of HIVST providers
Sub-national—Implementing partners	Fixed costs	None	
	Variable costs	R3. Personnel and Per diems—Headquarters Implementing partner	Total number of HIVST providers
Local—HIVST distribution areas	Fixed costs	None	
	Variable costs	R4. Personnel and Per diems—Field (HIVST distributors)	Total number of HIVST providers
		R5. HIV self-testing kits (implementation phase only)	Number of HIVST kits to distribute

* *The selection of scale variables was done in a way to account for the fact that the project is in early implementation phase (HIVST kits distribution targets not always reached by CSO in early phase) and the COVID-19 pandemic impact (reduced field activities), meaning CSO were not working at full capacity during the observed costing period. Therefore, the model uses predominantly the number of providers as scale up variable rather than the number of HIVST kits distributed during our observed period to limit the risks of bias. The number of kits to distribute is used to estimate projected costs based on HIVST volume distribution targets for each year 2021–2023*.

*IPO, International Partner Organisation*.

Where:

**C**: Total cost

**j:** inputs differentiating intervention levels—international, national, district, and community

**FC**_****j****_: Fixed cost (independent of S_j_) for fixed input *j* (e.g., building, personnel at central level)

**VC**_****j****_: Variable cost for input *j* (e.g., field personnel, HIVST kits)

**UC**_****j****_**:** Unit cost per variable inputs *j* for one output (the type of unit depends of each category): new staff to train, HIVST kits to distribute, etc.

**S**_****j****_: Scale variable for input *j* to reach desired number of outputs: number of new providers required for scale-up, total number of providers at scale-up, number of HIVST kits to distribute.

In anticipation of planned project scale-up by respective country ministries of health and post-ATLAS transition, we conducted a series of scenario analyses varying some of the key model parameters by country and by scale-up year, considering 2021 as a transition year, 2022 partial scale-up, and 2023 as full scale-up. Four potential scenarios are presented in Table 3. Logistical and contextual challenges with CSO-led delivery channels to criminalised KP, and current donors' commitments for funding, were noted to cause challenges leading to uncertainty related to the timely attainment of targets. We therefore anticipate that those programmatic objectives might not be reached. Accounting for this would provide more nuanced scale economies, and we applied different percentages for reaching targets—higher percentages in Mali, where more funding is already secured (*scenario 1*). IPO's goal to progressively disengage to promote local project ownership overtime was considered. Note that we still account for 15% of international costs in 2023 because we assume another coordination component will still exist (and incur costs) within the local health system at central level. Year 2023 would then represent what it costs for the country to support HIVST post-ATLAS (*scenario 2*). We also assessed the impact of optimising delivery channels by simplifying the model of partners/sub-partners and decreased CSO headquarter costs by 20%, which is reasonable to assume when evaluating interventions transitioning from pilot (ATLAS) to routine implementation phase (*scenario 3*) (35). Finally, we conducted country-specific simulations to account for varying HIVST kit cost for each year considering factors such as bulk buying, maritime provision instead of airways (except Mali), and integrating HIVST delivery chain with other health supplies (*scenario 4*). Finally, we combined all scenarios above to assess the global impact on average costs at scale per KP and scale-up year.

**Table 3:** Selected parameters for the scenario analysis of costs at scale-up in Côte d'Ivoire, Senegal, and Mali (baseline: all parameters at 100%).

	**Scenario 1**			**Scenario 2**			**Scenario 3**			**Scenario 4**		
	**Reaching HIVST distribution volume targets** ** (% of target achieved)**			**Progressive disengagement of IPO** ** (% reduction of IPO costs)**			**Implementing partners headquarter costs** ** (% reduction of IP costs)**			**HIVST kit cost based on volumes** ** (% reduction of original kit cost)**		
	**2021**	**2022**	**2023**	**2021**	**2022**	**2023**	**2021**	**2022**	**2023**	**2021**	**2022**	**2023**
Côte d'Ivoire	−25%	−25%	−30%	As in baseline	−50%	−85%	−20%	−20%	−20%	−9%	−9%	−9%
Senegal	−25%	−25%	−30%	As in baseline	−50%	−85%	−20%	−20%	−20%	−17%	−17%	−17%
Mali	−20%	−20%	−25%	As in baseline	−50%	−85%	−20%	−20%	−20%	−13%	−13%	−13%

*IPO, International Partner Organisation; IP, Implementing Partner*.

This study was approved by the London School of Hygiene and Tropical Medicine (n° 17141/RR/13198, 31st March 2019) WHO Ethic Research Committee (n°ERC0003181, 7th August 2019), and by three national ethic committees: Comité National d'Ethique des Sciences de la vie et de la Santé de Côte d'Ivoire (n°049-19/MSHP/CNESVS-kp, 28th May 2019), Comité National d'Ethique pour la Recherche en santé du Sénégal (n°SEN19/32, 26th July 2019), and Comité d'Ethique de la Faculté de Médecine de Pharmacie et d'Odonto-Stomatologie de l'Université des Sciences et des Techniques de Bamako au Mali (n°2019/88/CE/FMPOS, 14th August 2019).

## Results

### Programme Outcomes in Côte d'Ivoire, Senegal, and Mali

During the costing period, 51,028, 14,472, and 34,353 HIVST kits were distributed in Côte d'Ivoire, Senegal, and Mali through a total of 161, 48, and 191 peer educators, respectively. These volumes corresponded to 68% (Côte d'Ivoire), 103% (Senegal), and 42% (Mali) of planned targets. The average number of HIVST kits distributed was 7,290 (range: 1,295–16,513) across 7 CSO in Côte d'Ivoire, 3,618 (range: 422–7,193) across the main four models composed of 11 CSO in Senegal (CSO-Associations, CSO-Mobile clinics, CSO-independent distributors, and the public partner working with PWUD only), and 6,871 (range: 2,688–17,891) across 5 CSO in Mali. In Côte d'Ivoire, 66% of kits (*N* = 33,647) were distributed to FSW, 26% (*N* = 13,250) to MSM, and 8% (*N* = 4,131) to PWUD. In Senegal, 64% of kits (*N* = 9,338) were distributed to FSW, 31% (*N* = 4,472) to MSM, and 5% (*N* = 662) to PWUD. In Mali, 80% of kits (*N* = 27,528) were distributed to FSW, and 20% (*N* = 6,825) to MSM.

### Project Total Costs and Average Costs per Kit Distributed, Distribution Target

**Figure 2:**
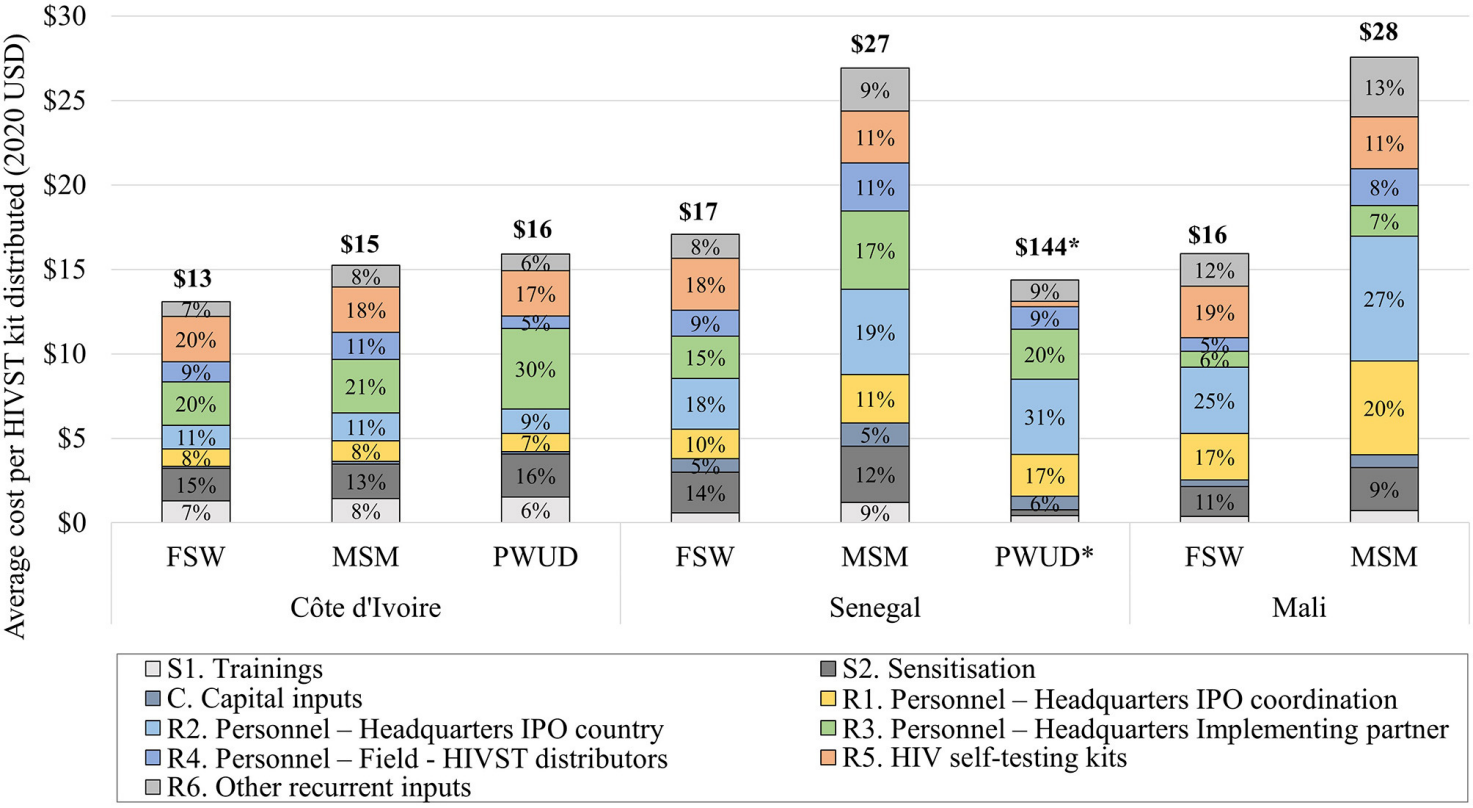
Average intervention costs by inputs for each key group—Côte d'Ivoire, Senegal, and Mali. *For PWUD in Senegal, costs are presented on this figure divided by 10 for scale purpose.

In Côte d'Ivoire, the total distribution costs were calculated as $440,648, $201,910, and $65,691 for FSW, MSM, and PWUD, respectively (Table 4). Start-up phase accounted for 25, 23, and 26% of total costs for FSW, MSM, and PWUD, respectively, while the development phase only accounted for 2% across key groups. Personnel costs at various intervention levels accounted for a substantial portion of total costs, at 47% for FSW, and 50% for MSM and PWUD, followed by HIVST kits costs at 20, 18, and 17% (Figure 2). Average cost per HIVST kit distributed were $13, $15, and $16 for FSW, MSM, and PWUD.

**Table 4:** Observed total and average intervention costs by intervention phase and key group—Côte d'Ivoire, Senegal, and Mali.

	**Côte d'Ivoire—Global estimates**					
	**FSW**		**MSM**		**PWUD**	
	**$**	**%**	**$**	**%**	**$**	**%**
**Intervention phases**						
Development	7,612	*2%*	3,518	*2%*	1,118	*2%*
Start–up (start–up and other costs)	120,874	*27%*	52,238	*26%*	18,687	*28%*
Implementation	312,162	*71%*	146,153	*72%*	45,887	*70%*
**Total annual costs**	**440,648**		**201,910**		**65,691**	
HIVST kits distributed	33,647		13,250		4,131	
**Average cost per HIVST kit distributed**	**13**		**15**		**16**	
	**Senegal—Global estimates**					
	**FSW**		**MSM**		**PWUD**	
	**$**	**%**	**$**	**%**	**$**	**%**
**Intervention phases**						
Development	8,262	*5%*	5,684	*5%*	4,754	*5%*
Start-up (start-up and other costs)	35,628	*22%*	25,579	*21%*	9,648	*10%*
Implementation	115,502	*72%*	89,111	*74%*	80,689	*85%*
**Total annual costs**	**159,393**		**120,374**		**95,091**	
HIVST kits distributed	9,338		4,472		662	
**Average cost per HIVST kit distributed**	**17**		**27**		**144**	
	**Mali—Global estimates**					
	**FSW**		**MSM**			
	**$**	**%**	**$**	**%**		
**Intervention phases**						
Development	11,544	*3%*	5,434	*3%*		
Start-up (start-up and other costs)	74,345	*17%*	29,633	*16%*		
Implementation	352,664	*80%*	153,093	*81%*		
**Total annual costs**	**438,553**		**188,159**			
HIVST kits distributed	27,528		6,825			
**Average cost per HIVST kit distributed**	**16**		**28**			

*HIVST, HIV Self-Testing kit; FSW, Female Sex workers; MSM, Men who have Sex with Men; PWUD, People who use drugs*.

For Senegal, total intervention costs were $159,393, $120,374, and $95,091 for FSW, MSM, and PWUD (Table 4). Start-up phase costs were 17% for FSW and MSM, and 5% for PWUD, and at a mean of 5% for development phase costs across groups. Personnel costs were 51%, 57%, and 78% of total costs while HIVST kits costs were 18%, 11%, and 2% for FSW, MSM, and PWUD, respectively (Figure 2). Average costs per kit were $17, $27, and $144 for FSW, MSM, and PWUD.

Finally, in Mali, total costs were $438,553 and $188,159 for FSW, and MSM (Table 4). Start-up phase and development phase costs accounted on average for 13% and 3% of total costs across groups. Personnel costs were 53%, and 61% of total costs, while HIVST kits costs were at 19% and 11% for FSW and MSM, respectively (Figure 2). Average cost per kit were $16 and $28 for FSW and MSM.

While the share of start-up costs as percentage of total costs was comparable between target groups in Côte d'Ivoire and in Mali, it differed in Senegal because the CSO delivering to PWUD were small organisations, hence being allocated a low share of start-up costs. Because the start-up period was longer in Côte d'Ivoire (6 months) compared to the one in Senegal and Mali (3 months), start-up costs as percentage of total costs were higher in Côte d'Ivoire.

Wide variations of average costs per HIVST kit distributed were found between CSO (Appendix Tables 2a–c). In Côte d'Ivoire, average cost per kit distributed ranged $9–$27 for FSW, $10–$29 for MSM, and only one CSO worked with PWUD. In Senegal, average costs were $13–$32 for FSW, $25–$28 for MSM, and $121–$156 for PWUD. In Mali, average cost per kit distributed ranged $15–$27 for FSW, and $17–$59 for MSM. In Senegal, CSO-Associations had lower average costs than CSO-Independent distributors (mean: $19 vs. $23), but overall distributed less HIVST kits (5,834 vs. 6,953 kits) to FSW and MSM.

The major driver of these cost differences both between and within key groups for all countries was the number of kits distributed per dispensing agent, except in Côte d'Ivoire where the average number of kits distributed per dispensing agent was comparable between groups. Another important driver of cost variation between and within groups for all countries was the total number of HIVST kits distributed by a CSO. An increase of any of these two drivers would lead to a reduction in average costs.

### Sensitivity Analysis of Costs Results

Appendix Figure 1 presents results from the univariate sensitivity analyses by key groups for Côte d'Ivoire (**1a**), Senegal (**1b**), and Mali (**1c**). Our unit costs per HIVST kit distributed remained robust when key cost parameters were varied. In Côte d'Ivoire, varying life of start-up sensitisation and training between 1 and 3 years had the strongest effect on costs ranging between $12–$17, $14–$19, and $14–$20 for FSW, MSM and PWUD, respectively. The life year of development and start-up phases, allocation factor swapping (for FSW and MSM) had a moderate effect with less than a dollar variation. The variation of discount rate almost had no effect on costs. In Senegal, the discount rate applied had the strongest effect with average costs varying between $17–$19, $26–$30, and $141–$163 for FSW, MSM, and PWUD, respectively due to higher proportion of capital costs compared to Côte d'Ivoire. Allocation factor swapping from trained distributors had an effect on average costs for PWUD (reduction to $127), while swapping from time-motion study results had no effect. In Mali, swapping of allocation factors has the strongest effect, but overall, average costs only varied by <2 dollars suggesting our average costs were quite robust.

Reaching HIVST distribution targets greatly reduced costs (not presented in Appendix Figure 1). Average cost per HIVST kit distributed were $9, $9, and $16 for FSW, MSM, and PWUD, assuming distribution targets were reached in Côte d'Ivoire. In Senegal, average costs per kit were $24, $23, and $47 for FSW, MSM, and PWUD assuming distribution targets were reached. Finally, in Mali, average cost per kit would be much lower if targets were reached, at $7 and $8 for FSW and MSM, respectively.

### Cost at Scale-Up Following National Strategic Plans

**Figure 3:**
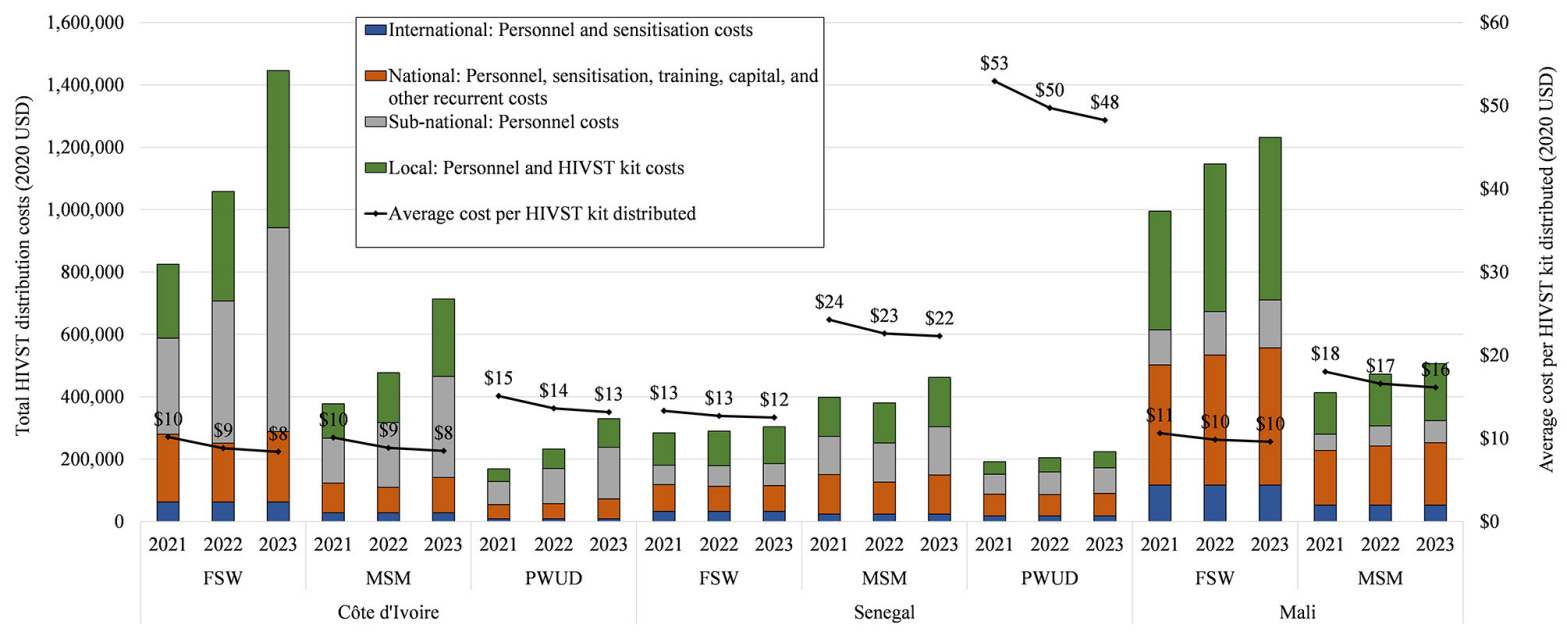
Total and average intervention costs in transition (2021) and at scale-up (2022–2023) by country and key population.

Costs at scale-up for each year of the National Strategic Plans are presented by country, year, and key groups in Figure 3, with details in Appendix Tables 3a–c.

Over the period 2021–2023, costs per kit distributed are on average at $9 (FSW and MSM), and $14 (PWUD) in Côte d'Ivoire; $13 (FSW), $23 (MSM) and $50 (PWUD) in Senegal; and $10 (FSW), and $17 (MSM) in Mali. We note the significant reduction of average costs at scale-up vs. observed average costs for FSW and MSM in Côte d'Ivoire, PWUD in Senegal, and all groups in Mali. Across countries, years, and key groups, the trend is an overall increase in total costs as expected. Although we estimate variation between countries and key groups, in transition and scale-up, overall cost drivers are fixed costs such as sensitisation activities, and headquarter-based personnel costs at national and sub-national level, and variable costs such as training and HIVST kits costs (varying with HIVST distribution targets). In Senegal, we estimate higher personnel costs at CSO level (headquarter- and field-based).

### Scenario Analysis of Scale-Up Costs

As the scale-up model does not account for other contextual factors related to the transition post-ATLAS, analyses of plausible scale-up scenario are presented in Appendix Figures 2a–c.

For all countries and key groups, we find that HIVST volumes are the major determinants of costs per HIVST kit distributed (economies of scale), followed by IPO withdrawal starting in 2022, reduction of implementers' central costs, and the estimated reduction of HIVST kit price. Accounting for all these factors together would increase estimated scale-up average costs between $9 (FSW−2023) and $18 (PWUD−2021) in Côte d'Ivoire, from $12 (FSW−2023) to $65 (PWUD−2021) in Senegal, and from $9 (FSW−2023) to $21 (MSM−2021) in Mali.

## Discussion

In this study, we estimated the cost of implementing HIVST for KP and their partners in three West African countries. Across countries, we found that costs ranged between $13-$17 for FSW, $15-$28 for MSM and $16-$144 for PWUD. Note that PWUD channels distribute small quantities of HIVST kits, and average costs are therefore highly sensitive to scale of operation between CSO. Major cost contributors were personnel costs at central and regional intervention levels. Start-up costs across countries, corresponding to sensitisation of CSO and other partners, and training costs contributed to 10–28% of total costs. This is due to the complexity and lengthy process of building partnerships with numerous local CSO and involving key stakeholders in an intervention fully integrated with existing health care delivery services for KP. Costs per kit distributed were lowest in Côte d'Ivoire and highest in Senegal. Across countries, average costs per HIVST were lowest for FSW, followed by MSM, then PWUD. These differences could be explained by HIVST volumes by channels with a total of 70,513 kits distributed to FSW, 24,547 kits to MSM, and 4,793 kits to PWUD during our costing period. However, it is likely that other factors played a role. For instance, in Senegal and Mali, several episodes of violence against MSM were reported at different time points (unrelated to the programme), and CSO had to suspend their field activities for security reasons, contributing to an unstable, and therefore costly, delivery system of kits for this group. In Mali, there were safety concerns due to the country's *Coup d'Etat* in August 2020, and ongoing armed conflict with intermittent suspension of fieldwork activities. Indeed, estimated average costs per kit would be as low as $7 (FSW) and $8 (MSM) assuming targets were reached in Mali. Finally, the COVID-19 pandemic also led to reduced (Côte d'Ivoire and Mali) or suspended (Senegal) activities during 2–3 months, leading to high observed costs, although self-testing was shown to be a timely alternative to provider-delivered HIV testing during periods of lockdown and reduced social interactions (36).

Important average costs variations between CSO were observed. High number of kits distributed per dispensing agent led to a reduction in average costs and depended on the type of HIVST distribution activity with high distribution in bars and brothels, and low distribution in small gatherings at KP's house. CSO-specific policy with monthly maximum targets of kits distribution per agent could potentially lead to higher average costs. Small number of HIVST kits distributed per CSO was also driving average costs high and was explained by the type of population reached (e.g., CSO working with PWUD only deliver small HIVST volumes), and the CSO size. To a lesser extent in Mali, numerous HIVST delivery models per CSO (some not presented here such as Index and STI services) could lead to higher spreading of central costs across models, and therefore, a reduction of average costs.

Our costs were comparable to other community-based HIVST costing studies, many of them arising from the STAR (*HIV Self-Testing AfRica*) project (37, 38)^1^. Across six southern Africa countries (Malawi, Zambia, Zimbabwe, South Africa, Lesotho, eSwatini), costs per kit distributed ranged from $8 for door-to-door distribution in Malawi to $18 for mobile integration (more similar to the ATLAS programme) in South Africa (25, 26, 39, 40). Although HIVST volumes were generally higher as targeting the general population and benefiting from economies of scale, many of these models were highly vertical incurring significant above service level costs. However, cost per kit distributed to South African FSW and MSM were lower than our observed costs at $4 and $6, respectively, for 19,901 and 12,218 kits distributed. This is partly explained by the high number of HIVST delivery models in South Africa and sharing of central costs across models (39). Additionally, our costs were comparable to one study in Côte d'Ivoire reporting HTS unit costs from the Ivorian *Programme National de Lutte contre le Sida (PNLS)* for FSW and MSM at $16 and $21, respectively (41). However, one should consider the reduced costs to the kit user (in terms of transportation cost or opportunity cost for example), and therefore to society, when comparing community-based HIVST distribution and facility-based provider-delivered HTS costs (42, 43).

The scale-up model suggests that these early-stage CSO-led community-based HIVST distribution programmes can exhibit economies of scale. When comparing year 2023 with observed costs, we estimated variable scale economies between groups and countries, with about 56% (FSW), 63% (MSM), and 10% (PWUD) of average cost reduction in Côte d'Ivoire, 19% (FSW), 12% (MSM), and 66% (PWUD) in Senegal, and 35% (FSW), 41% (MSM) in Mali. Beyond scale economies, other contextual factors were considered, such as accounting for progressive integration of the ATLAS project to existing CSO and withdrawal of the IPO. The scenario analysis suggests that, overall, even if target were not reached, costs at scale would decrease in Côte d'Ivoire (except PWUD) and Mali. However, results are more nuanced for Senegal with constant (FSW) or increasing average costs (MSM, PWUD) due to high fixed costs at sub-national level.

Our study has several limitations. First, our outcome metric “per HIVST kit distributed” does not fully capture the HIVST cascade. For example, there remain uncertainties related to the true percentage of kits use, the actual final users of the kit (e.g., HIVST distribution through a FSW model could also be used by their clients), and among those with a reactive HIVST the linkage rate to confirmatory testing. However, there is now large evidence on high acceptability of HIVST kits in the general population and among KP (11, 13, 14, 17, 18, 44–47). Moreover, the ATLAS programme is currently trying to evaluate the impact of HIVST on HIV case finding and ART initiation, these data will then feed in a modelling analysis to estimate cost-effectiveness. Second, total and average costs are estimated across a diverse range of CSO for each country leading to inevitable cost variation by distribution channel. Third, the COVID-19 pandemic led to reduced/suspended activities during a trimester for some CSO, but also encouraged the use of HIVST by other actors as a timely alternative to HTS in response to lockdown and social distancing, therefore, its impact on costs and project outcomes is difficult to assess (36). Fourth, scale-up costs and scenario analysis were conducted in collaboration with the implementer to ensure model assumptions were close to reality, but these remain arbitrary and should be interpreted with caution.

In three countries of West Africa, HIVST kit provision to KP through CSO had higher initial costs during the study period, related to the progressive integration of HIVST to CSO activities, and a challenging implementing environment (criminalised KP, pandemic COVID-19, security concerns). The analysis of costs at scale suggests that, in transition to scale-up and further integration of the ATLAS project, this model shows large potential for substantial economies of scale as programmes scale-up and mature.

Recent modelling studies in Cameroon, Senegal, Côte d'Ivoire, and South Africa show that key populations and their sexual partners, particularly FSW and their clients, can play an important role in HIV transmission in both low and high HIV prevalence settings due to prevention gaps (3, 4, 48). HIV prevention and treatment strategies targeting these groups are essential for controlling the HIV epidemic and are likely to provide good value for money. The CSO-led HIVST delivery model is particularly relevant as it remains today the most promising strategy for reaching KP, their sexual partners and clients of FSW not accessing HIV testing, so-called “hidden populations.” Further research will assess the overall cost-effectiveness of the CSO-led HIVST delivery programme.

## Data Availability Statement

The raw data supporting the conclusions of this article will be made available by the authors, without undue reservation.

## Ethics Statement

The studies involving human participants were reviewed and approved by the London School of Hygiene and Tropical Medicine Research Committee (n° 17141/RR/13198, 31st March 2019) WHO Ethic Research Committee (n°ERC0003181, 7th August 2019), and by three national ethic committees: Comité National d'Ethique des Sciences de la vie et de la Santé de Côte d'Ivoire (n°049-19/MSHP/CNESVS-kp, 28th May 2019), Comité National d'Ethique pour la Recherche en santé du Sénégal (n°SEN19/32, 26th July 2019), and Comité d'Ethique de la Faculté de Médecine de Pharmacie et d'Odonto-Stomatologie de l'Université des Sciences et des Techniques de Bamako au Mali (n°2019/88/CE/FMPOS, 14th August 2019). The patients/participants provided their written informed consent to participate in this study.

## Composition of the Atlas Team

### ATLAS Research Team

Amani Elvis Georges (Programme PACCI, ANRS Research Site, Treichville University Hospital, Abidjan, Côte d'Ivoire); Badiane Kéba (Solthis, Sénégal); Bayac Céline (Solthis, France); Bekelynck Anne (Programme PACCI, ANRS Research Site, Treichville University Hospital, Abidjan, Côte d'Ivoire); Boily Marie-Claude (Department of Infectious Disease Epidemiology, Medical Research Council Centre for Global Infectious Disease Analysis, Imperial College London, London, United Kingdom); Boye Sokhna (Centre Population et Développement, Institut de Recherche pour le Développement, Université Paris Descartes, Inserm, Paris, France); Breton Guillaume (Solthis, Paris, France); d'Elbée Marc (Department of Global Health and Development, Faculty of Public Health and Policy, London School of Hygiene and Tropical Medicine, London, United Kingdom); Desclaux Alice (Institut de Recherche pour le Développement, Transvihmi (UMI 233 IRD, 1175 INSERM, Montpellier University), Montpellier, France/CRCF, Dakar, Sénégal); Desgrées du Loû Annabel (Centre Population et Développement, Institut de Recherche pour le Développement, Université Paris Descartes, Inserm, Paris, France); Diop Papa Moussa (Solthis, Sénégal); Ehui Eboi (Directeur Coordonnateur, PNLS; Graham Medley, Department of Global Health and Development, Faculty of Public Health and Policy, London School of Hygiene and Tropical Medicine, London, United Kingdom); Jean Kévin (Laboratoire MESuRS, Conservatoire National des Arts et Métiers, Paris, France); Keita Abdelaye (Institut National de Recherche en Santé Publique, Bamako, Mali); Kouassi Kra Arsène (Centre Population et Développement, Institut de Recherche pour le Développement, Université Paris Descartes, Inserm, Paris, France); Ky-Zerbo Odette (TransVIHMI, IRD, Université de Montpellier, INSERM); Larmarange Joseph (Centre Population et Développement, Institut de Recherche pour le Développement, Université Paris Descartes, Inserm, Paris, France); Maheu-Giroux Mathieu (Department of Epidemiology, Biostatistics, and Occupational Health, School of Population and Global Health, McGill University, Montréal, QC, Canada); Moh Raoul (1. Programme PACCI, ANRS Research Site, Treichville University Hospital, Abidjan, Côte d'Ivoire, 2. Department of Infectious and Tropical Diseases, Treichville University Teaching Hospital, Abidjan, Côte d'Ivoire, 3. Medical School, University Felix Houphouet Boigny, Abidjan, Côte d'Ivoire); Mosso Rosine (ENSEA Ecole Nationale de Statistiques et d'Economie Appliquée, Abidjan, Côte d'Ivoire); Ndour Cheikh Tidiane (Division de Lutte contre le Sida et les IST, Ministère de la Santé et de l'Action Sociale Institut d'Hygiène Sociale, Dakar, Sénégal); Paltiel David (Yale School of Public Health, New Haven, CT, United States); Pourette Dolorès (Centre Population et Développement, Institut de Recherche pour le Développement, Université Paris Descartes, Inserm, Paris, France); Rouveau Nicolas (Centre Population et Développement, Institut de Recherche pour le Développement, Université Paris Descartes, Inserm, Paris, France); Silhol Romain (Medical Research Council Centre for Global Infectious Disease Analysis, Department of Infectious Disease Epidemiology, Imperial College London, London, United Kingdom); Simo Fotso Arlette (Centre Population et Développement, Institut de Recherche pour le Développement, Université Paris Descartes, Inserm, Paris, France); Terris-Prestholt Fern (Department of Global Health and Development, Faculty of Public Health and Policy, London School of Hygiene and Tropical Medicine, London, United Kingdom); Traore Métogara Mohamed (Solthis, Côte d'Ivoire).

### Solthis Coordination Team

Diallo Sanata (Solthis, Dakar, Sénégal); Doumenc (Aïdara Clémence-Solthis, Dakar, Sénégal); Geoffroy Olivier (Solthis, Abidjan, Côte d'Ivoire); Kabemba Odé Kanku (Solthis, Bamako, Mali); Vautier Anthony (Solthis, Dakar, Sénégal).

### Implementation in Côte d'Ivoire

Abokon Armand (Fondation Ariel Glaser, Côte d'Ivoire); Anoma Camille (Espace Confiance, Côte d'Ivoire); Diokouri Annie (Fondation Ariel Glaser, Côte d'Ivoire); Kouame Blaise (Service Dépistage, PNLS); Kouakou Venance (Heartland Alliance, Côte d'Ivoire); Koffi Odette (Aprosam, Côte d'Ivoire); Kpolo Alain (Michel-Ruban Rouge, Côte d'Ivoire); Tety Josiane (Blety, Côte d'Ivoire); Traore Yacouba (ORASUR, Côte d'Ivoire).

### Implementation in Mali

Bagendabanga Jules (FHI 360, Mali); Berthé Djelika (PSI, Mali); Diakite Daouda (Secrétariat Exécutif du Haut Conseil National de Lutte contre le Sida, Mali); Diakité Mahamadou (Danayaso, Mali); Diallo Youssouf (CSLS/MSHP); Daouda Minta (Comité scientifique VIH); Hessou Septime (Plan Mali); Kanambaye Saidou (PSI, Mali); Kanoute Abdul Karim (Plan Mali); Keita Dembele Bintou (Arcad-Sida, Mali); Koné Dramane (Secrétariat Exécutif du Haut Conseil National de Lutte contre le Sida, Mali); Koné Mariam (AKS, Mali); Maiga Almoustapha (Comité scientifique VIH; Nouhoum Telly, CSLS/MSHP); Saran Keita Aminata (Soutoura, Mali); Sidibé Fadiala (Soutoura, Mali); Tall Madani (FHI 360, Mali); Yattassaye Camara Adam (Arcad-Sida, Mali); Sanogo Abdoulaye (Amprode Sahel, Mali).

### Implementation in Senegal

Bâ Idrissa (CEPIAD, Sénégal); Diallo Papa Amadou Niang (CNLS, Sénégal); Fall Fatou (DLSI, Ministère de la Santé et de l'action sociale, Sénégal); Guèye NDèye Fatou NGom (CTA, Sénégal); Ndiaye Sidy Mokhtar (Enda Santé, Sénégal); Niang Alassane Moussa (DLSI, Ministère de la Santé et de l'action sociale, Sénégal); Samba Oumar (CEPIAD, Sénégal); Thiam Safiatou (CNLS, Sénégal); Turpin Nguissali M.E. (nda Santé, Sénégal).

### Partners

Bouaré Seydou (Assistant de recherche, Mali); Camara Cheick Sidi (Assistant de recherche, Mali); Kouadio Brou Alexis (Assistant de recherche, Côte d'Ivoire); Sarrassat Sophie (Centre for Maternal, Adolescent, Reproductive and Child Health, London School of Hygiene and Tropical Medicine, London, United kingdom); Sow Souleyman (Assistant de recherche, Sénégal).

## Author Contributions

Md'E and FT-P designed the study. Md'E coordinated, conducted data analysis, and wrote the paper. MT and KB conducted data collection/analysis. AV, AF, OK, NR, PG-F, MM-G, M-CB, GM, and JL provided logistical support and intellectual inputs. All authors revised the manuscript and agreed for publication.

## Conflict of Interest

The authors declare that the research was conducted in the absence of any commercial or financial relationships that could be construed as a potential conflict of interest.

## Footnotes

^1^Ahmed N, Terris-Prestholt F, Ong JJ, d'Elbée M, Rotolo S, Johnson C, et al. A systematic literature review of costs and cost-effectiveness analyses of HIV testing services in sub-Saharan Africa.
