# Supplementary material for: Costs and Scale-Up Costs of Integrating HIV Self-Testing Into Civil Society Organisation-Led Programmes for Key Populations in Côte d'Ivoire, Senegal, and Mali
Source: Front Public Health. 2021 May 24;9:653612. doi: 10.3389/fpubh.2021.653612 (PMC8182047; doi:10.3389/fpubh.2021.653612)
Supplement: Supplementary file 1 [file Data_Sheet_1.docx]

# Supplementary Material

**Appendix Table 1.** Allocation factors for the top-down costing analysis by input type

|  | **Allocation factors to site level** | | |
| --- | --- | --- | --- |
| **Input type** | **Côte d’Ivoire** | **Senegal** | **Mali** |
| *Start-up costs* |  |  |  |
| S1. Trainings | % trained distributors | % trained distributors | % trained distributors |
| S2. Sensitisation | % of cohort size | % of cohort size | % of cohort size |
| *Capital costs* |  |  |  |
| C1. Buildings and storage | % direct expenditure | % direct expenditure | % direct expenditure |
| C2. Equipment | % direct expenditure | % direct expenditure | % direct expenditure |
| C3. Vehicles | % HIVST kits distributed | % HIVST kits distributed | % HIVST kits distributed |
| C4. Other capital costs | % direct expenditure | % direct expenditure | % direct expenditure |
| *Recurrent costs* |  |  |  |
| R1. Personnel & Per diems – Headquarters IPO coordination | % trained distributors | Equally shared across sites | % trained distributors |
| R2. Personnel & Per diems – Headquarters IPO country | % trained distributors | Equally shared across sites | % trained distributors |
| R3. Personnel & Per diems – Headquarters Implementing partner | % trained distributors | % HIVST distributors | % trained distributors |
| R4. Personnel & Per diems – Field - HIVST distributors | % trained distributors | % HIVST distributors | % trained distributors |
| R5. HIV self-testing kits | % HIVST kits distributed | % HIVST kits distributed | % HIVST kits distributed |
| R6. Vehicle operation and maintenance/transportation | % HIVST kits distributed | % HIVST kits distributed | % HIVST kits distributed |
| R7. Building operation and maintenance | % direct expenditure | % direct expenditure | % direct expenditure |
| R8. Other recurrent costs | % direct expenditure | % direct expenditure | % direct expenditure |

IPO: International Partner Organisation

**Appendix Table 2.a.** Observed total and average intervention costs by CSO and key groups – Côte d’Ivoire (1/2)

|  | **CSO1** | | | | **CSO2** | | **CSO3** | | | | | | **CSO4** | |
| --- | --- | --- | --- | --- | --- | --- | --- | --- | --- | --- | --- | --- | --- | --- |
|  | **FSW** | | **MSM** | | **FSW** | | **FSW** | | **PWUD** | | **MSM** | | **MSM** | |
|  | **$** | **%** | **$** | **%** | **$** | **%** | **$** | **%** | **$** | **%** | **$** | **%** | **$** | **%** |
| **INTERVENTION PHASES** |  |  |  |  |  |  |  |  |  |  |  |  |  |  |
| Development | 1,327 | *2%* | 543 | *2%* | 1,766 | *2%* | 1,941 | *2%* | 1,118 | *2%* | 721 | *1%* | 634 | *3%* |
| Start-up (start-up and other costs) | 21,890 | *27%* | 8,887 | *31%* | 24,169 | *24%* | 33,812 | *33%* | 18,687 | *28%* | 13,436 | *26%* | 5,581 | *23%* |
| Implementation | 58,166 | *71%* | 19,497 | *67%* | 74,030 | *74%* | 67,769 | *65%* | 45,887 | *70%* | 37,774 | *73%* | 18,337 | *75%* |
| **COST CATEGORIES** |  |  |  |  |  |  |  |  |  |  |  |  |  |  |
| **Start-up** |  |  |  |  |  |  |  |  |  |  |  |  |  |  |
| S1. Trainings | 7,379 | 9% | 2,635 | 9% | 10,541 | 11% | 11,068 | 11% | 6,324 | 10% | 3,689 | 7% | 3,689 | 15% |
| S2. Sensitisation | 12,256 | 15% | 5,290 | 18% | 10,684 | 11% | 19,479 | 19% | 10,475 | 16% | 8,493 | 16% | 816 | 3% |
| *Total Start-up* | *19,634* | *24%* | *7,925* | *27%* | *21,225* | *21%* | *30,547* | *30%* | *16,799* | *26%* | *12,183* | *23%* | *4,505* | *18%* |
| **Capital** |  |  |  |  |  |  |  |  |  |  |  |  |  |  |
| C1. Buildings and storage | 576 | 1% | 187 | 1% | 971 | 1% | 462 | 0% | 426 | 1% | 239 | 0% | 372 | 2% |
| C2. Equipment | 145 | 0% | 54 | 0% | 285 | 0% | 198 | 0% | 147 | 0% | 72 | 0% | 86 | 0% |
| C3. Vehicles | 34 | 0% | 34 | 0% | 11 | 0% | 34 | 0% | 23 | 0% | 34 | 0% | 11 | 0% |
| C4. Other capital costs | 10 | 0% | 4 | 0% | 22 | 0% | 14 | 0% | 11 | 0% | 5 | 0% | 7 | 0% |
| *Total Capital* | *766* | *1%* | *278* | *1%* | *1,289* | *1%* | *708* | *1%* | *606* | *1%* | *350* | *1%* | *476* | *2%* |
| **Recurrent** |  |  |  |  |  |  |  |  |  |  |  |  |  |  |
| R1. Personnel – Headquarters IPO coordination | 4,408 | 5% | 1,574 | 5% | 9,347 | 9% | 5,946 | 6% | 4,455 | 7% | 2,021 | 4% | 3,128 | 13% |
| R2. Personnel – Headquarters IPO country | 5,862 | 7% | 2,093 | 7% | 12,704 | 13% | 7,845 | 8% | 5,984 | 9% | 2,671 | 5% | 4,243 | 17% |
| R3. Personnel – Headquarters IP | 15,686 | 19% | 4,886 | 17% | 19,094 | 19% | 28,644 | 28% | 19,665 | 30% | 19,101 | 37% | 3,826 | 16% |
| R4. Personnel – Field - HIVST distributors | 5,599 | 7% | 1,756 | 6% | 7,243 | 7% | 3,230 | 3% | 3,055 | 5% | 1,638 | 3% | 2,424 | 10% |
| R5. HIV self-testing kits | 25,296 | 31% | 8,076 | 28% | 22,166 | 22% | 21,730 | 21% | 11,068 | 17% | 11,446 | 22% | 3,470 | 14% |
| R6. Vehicle operation and maintenance | 1,276 | 2% | 1,276 | 4% | 653 | 1% | 1,134 | 1% | 1,019 | 2% | 1,159 | 2% | 622 | 3% |
| R7. Building operation and maintenance | 2,080 | 3% | 774 | 3% | 4,586 | 5% | 2,716 | 3% | 2,224 | 3% | 990 | 2% | 1,364 | 6% |
| R8. Other recurrent costs | 775 | 1% | 288 | 1% | 1,657 | 2% | 1,024 | 1% | 816 | 1% | 372 | 1% | 494 | 2% |
| *Total Recurrent* | *60,983* | *75%* | *20,724* | *72%* | *77,450* | *77%* | *72,268* | *70%* | *48,286* | *74%* | *39,399* | *76%* | *19,571* | *80%* |
| **TOTAL ANNUAL COSTS** | **81,383** |  | **28,928** |  | **99,964** |  | **103,523** |  | **65,691** |  | **51,931** |  | **24,552** |  |
| HIVST kits distributed | 9,441 |  | 3,014 |  | 8,273 |  | 8,110 |  | 4,131 |  | 4,272 |  | 1,295 |  |
| **Average cost per HIVST kit distributed** | **9** |  | **10** |  | **12** |  | **13** |  | **16** |  | **12** |  | **19** |  |

CSO: Civil Society Organisation, IPO: International Partner Organisation, IP: Implementing Partner, HIVST: HIV Self-Testing kit, FSW: Female Sex workers, MSM: Men who have Sex with Men, PWUD: People who use drugs

**Appendix Table 2.a.** Observed total and average intervention costs by CSO and key groups – Côte d’Ivoire (2/2)

|  | **CSO5** | | | | **CSO6** | | | | **CSO7** | | | |
| --- | --- | --- | --- | --- | --- | --- | --- | --- | --- | --- | --- | --- |
|  | **FSW** | | **MSM** | | **FSW** | | **MSM** | | **FSW** | | **MSM** | |
|  | **$** | **%** | **$** | **%** | **$** | **%** | **$** | **%** | **$** | **%** | **$** | **%** |
| **INTERVENTION PHASES** |  |  |  |  |  |  |  |  |  |  |  |  |
| Development | 554 | *1%* | 466 | *2%* | 1,166 | *2%* | 729 | *2%* | 858 | *2%* | 424 | *2%* |
| Start-up (start-up and other costs) | 7,090 | *18%* | 5,240 | *17%* | 18,641 | *27%* | 10,576 | *25%* | 15,271 | *33%* | 8,518 | *35%* |
| Implementation | 31,650 | *81%* | 24,402 | *81%* | 50,042 | *72%* | 31,008 | *73%* | 30,505 | *65%* | 15,135 | *63%* |
| **COST CATEGORIES** |  |  |  |  |  |  |  |  |  |  |  |  |
| **Start-up** |  |  |  |  |  |  |  |  |  |  |  |  |
| S1. Trainings | 3,162 | 8% | 2,635 | 9% | 6,852 | 10% | 4,216 | 10% | 4,743 | 10% | 2,108 | 9% |
| S2. Sensitisation | 2,990 | 8% | 1,811 | 6% | 9,842 | 14% | 5,133 | 12% | 9,070 | 19% | 5,670 | 24% |
| *Total Start-up* | *6,152* | *16%* | *4,447* | *15%* | *16,694* | *24%* | *9,349* | *22%* | *13,813* | *30%* | *7,778* | *32%* |
| **Capital** |  |  |  |  |  |  |  |  |  |  |  |  |
| C1. Buildings and storage | 163 | 0% | 130 | 0% | 364 | 1% | 219 | 1% | 302 | 1% | 146 | 1% |
| C2. Equipment | 105 | 0% | 84 | 0% | 241 | 0% | 144 | 0% | 143 | 0% | 69 | 0% |
| C3. Vehicles | 11 | 0% | 11 | 0% | 11 | 0% | 11 | 0% | 23 | 0% | 23 | 0% |
| C4. Other capital costs | 9 | 0% | 7 | 0% | 20 | 0% | 12 | 0% | 11 | 0% | 5 | 0% |
| *Total Capital* | *287* | *1%* | *232* | *1%* | *636* | *1%* | *387* | *1%* | *479* | *1%* | *243* | *1%* |
| **Recurrent** |  |  |  |  |  |  |  |  |  |  |  |  |
| R1. Personnel – Headquarters IPO coordination | 3,464 | 9% | 2,887 | 10% | 7,506 | 11% | 4,619 | 11% | 4,206 | 9% | 1,869 | 8% |
| R2. Personnel – Headquarters IPO country | 4,749 | 12% | 3,957 | 13% | 10,289 | 15% | 6,332 | 15% | 5,717 | 12% | 2,541 | 11% |
| R3. Personnel – Headquarters IP | 6,451 | 16% | 4,938 | 16% | 10,096 | 14% | 6,306 | 15% | 6,566 | 14% | 3,017 | 13% |
| R4. Personnel – Field - HIVST distributors | 9,682 | 25% | 6,918 | 23% | 11,359 | 16% | 7,252 | 17% | 2,710 | 6% | 1,244 | 5% |
| R5. HIV self-testing kits | 5,305 | 14% | 3,990 | 13% | 6,950 | 10% | 3,957 | 9% | 8,705 | 19% | 4,563 | 19% |
| R6. Vehicle operation and maintenance | 816 | 2% | 816 | 3% | 816 | 1% | 816 | 2% | 1,305 | 3% | 1,305 | 5% |
| R7. Building operation and maintenance | 1,759 | 4% | 1,417 | 5% | 4,054 | 6% | 2,428 | 6% | 2,302 | 5% | 1,115 | 5% |
| R8. Other recurrent costs | 629 | 2% | 507 | 2% | 1,450 | 2% | 868 | 2% | 832 | 2% | 403 | 2% |
| *Total Recurrent* | *32,855* | *84%* | *25,429* | *84%* | *52,520* | *75%* | *32,578* | *77%* | *32,343* | *69%* | *16,057* | *67%* |
| **TOTAL ANNUAL COSTS** | **39,294** |  | **30,108** |  | **69,850** |  | **42,314** |  | **46,635** |  | **24,078** |  |
| HIVST kits distributed | 1,980 |  | 1,489 |  | 2,594 |  | 1,477 |  | 3,249 |  | 1,703 |  |
| **Average cost per HIVST kit distributed** | **20** |  | **20** |  | **27** |  | **29** |  | **14** |  | **14** |  |

CSO: Civil Society Organisation, IPO: International Partner Organisation, IP: Implementing Partner, HIVST: HIV Self-Testing kit, FSW: Female Sex workers, MSM: Men who have Sex with Men, PWUD: People who use drugs

**Appendix Table 2.b.** Observed total and average intervention costs by CSO and key groups – Senegal

|  | **CSO - Associations** | | | | **CSO – Mobile clinic** | | **CSO – Independent distributors** | | | | | | **Public partner** | |
| --- | --- | --- | --- | --- | --- | --- | --- | --- | --- | --- | --- | --- | --- | --- |
|  | **MSM** | | **FSW** | | **FSW** | | **MSM** | | **FSW** | | **PWUD** | | **PWUD** | |
|  | **$** | **%** | **$** | **%** | **$** | **%** | **$** | **%** | **$** | **%** | **$** | **%** | **$** | **%** |
| **INTERVENTION PHASES** |  |  |  |  |  |  |  |  |  |  |  |  |  |  |
| Development | 2,689 | *5%* | 2,644 | *5%* | 2,575 | *8%* | 2,996 | *5%* | 3,043 | *4%* | 2,176 | *7%* | 2,578 | *4%* |
| Start-up (start-up and other costs) | 12,097 | *22%* | 9,437 | *19%* | 7,154 | *22%* | 13,482 | *21%* | 19,037 | *24%* | 3,295 | *11%* | 6,353 | *10%* |
| Implementation | 41,122 | *74%* | 36,705 | *75%* | 23,056 | *70%* | 47,989 | *74%* | 55,742 | *72%* | 23,585 | *81%* | 57,104 | *86%* |
| **COST CATEGORIES** |  |  |  |  |  |  |  |  |  |  |  |  |  |  |
| **Start-up** |  |  |  |  |  |  |  |  |  |  |  |  |  |  |
| S1. Trainings | 3,890 | 7% | 2,947 | 6% | 1,022 | 3% | 1,532 | 2% | 1,532 | 2% | 511 | 2% | 2,240 | 3% |
| S2. Sensitisation | 5,628 | 10% | 3,939 | 8% | 3,684 | 11% | 9,209 | 14% | 14,734 | 19% | 614 | 2% | 1,663 | 3% |
| *Total Start-up* | *9,517* | *17%* | *6,886* | *14%* | *4,705* | *14%* | *10,741* | *17%* | *16,267* | *21%* | *1,125* | *4%* | *3,902* | *6%* |
| **Capital** |  |  |  |  |  |  |  |  |  |  |  |  |  |  |
| C1. Buildings and storage | 1,990 | 4% | 1,450 | 3% | 1,758 | 5% | 3,886 | 6% | 4,077 | 5% | 1,211 | 4% | 4,024 | 6% |
| C2. Equipment | 64 | 0% | 48 | 0% | 59 | 0% | 125 | 0% | 131 | 0% | 39 | 0% | 124 | 0% |
| C3. Vehicles | 61 | 0% | 53 | 0% | 19 | 0% | 36 | 0% | 36 | 0% | 12 | 0% | 34 | 0% |
| C4. Other capital costs | 0 | 0% | 0 | 0% | 0 | 0% | 0 | 0% | 0 | 0% | 0 | 0% | 0 | 0% |
| *Total Capital* | *2,115* | *4%* | *1,551* | *3%* | *1,836* | *6%* | *4,048* | *6%* | *4,244* | *5%* | *1,262* | *4%* | *4,183* | *6%* |
| **Recurrent** |  |  |  |  |  |  |  |  |  |  |  |  |  |  |
| R1. Personnel – Headquarters IPO coordination | 6,414 | 11% | 5,231 | 11% | 4,639 | 14% | 6,414 | 10% | 6,414 | 8% | 6,414 | 22% | 9,963 | 15% |
| R2. Personnel – Headquarters IPO country | 11,275 | 20% | 8,962 | 18% | 7,805 | 24% | 11,275 | 17% | 11,275 | 14% | 11,275 | 39% | 18,216 | 28% |
| R3. Personnel – Headquarters IP | 9,012 | 16% | 6,759 | 14% | 4,882 | 15% | 11,716 | 18% | 11,716 | 15% | 3,905 | 13% | 15,578 | 24% |
| R4. Personnel – Field - HIVST distributors | 5,512 | 10% | 4,134 | 8% | 2,986 | 9% | 7,166 | 11% | 7,166 | 9% | 2,389 | 8% | 6,475 | 10% |
| R5. HIV self-testing kits | 6,776 | 12% | 11,176 | 23% | 3,148 | 10% | 6,985 | 11% | 14,410 | 19% | 739 | 3% | 1,299 | 2% |
| R6. Vehicle operation and maintenance | 3,105 | 6% | 2,511 | 5% | 885 | 3% | 1,863 | 3% | 1,863 | 2% | 621 | 2% | 1,955 | 3% |
| R7. Building operation and maintenance | 788 | 1% | 580 | 1% | 707 | 2% | 1,540 | 2% | 1,615 | 2% | 480 | 2% | 1,572 | 2% |
| R8. Other recurrent costs | 1,392 | 2% | 996 | 2% | 1,191 | 4% | 2,719 | 4% | 2,852 | 4% | 847 | 3% | 2,893 | 4% |
| *Total Recurrent* | *44,275* | *79%* | *40,349* | *83%* | *26,243* | *80%* | *49,677* | *77%* | *57,311* | *74%* | *26,669* | *92%* | *57,951* | *88%* |
| **TOTAL ANNUAL COSTS** | **55,908** |  | **48,786** |  | **32,785** |  | **64,466** |  | **77,822** |  | **29,056** |  | **66,036** |  |
| HIVST kits distributed | 2,202 |  | 3,632 |  | 1,023 |  | 2,270 |  | 4,683 |  | 240 |  | 422 |  |
| **Average cost per HIVST kit distributed** | **25** |  | **13** |  | **32** |  | **28** |  | **17** |  | **121** |  | **156** |  |

CSO: Civil Society Organisation, IPO: International Partner Organisation, IP: Implementing Partner, HIVST: HIV Self-Testing kit, FSW: Female Sex workers, MSM: Men who have Sex with Men, PWUD: People who use drugs

**Appendix Table 2.c.** Observed total and average intervention costs by CSO and key groups – Mali (1/2)

|  | **CSO1** | | | | **CSO2** | | | |
| --- | --- | --- | --- | --- | --- | --- | --- | --- |
|  | **MSM** | | **FSW** | | **MSM** | | **FSW** | |
|  | **$** | **%** | **$** | **%** | **$** | **%** | **$** | **%** |
| **INTERVENTION PHASES** |  |  |  |  |  |  |  |  |
| Development | 1,245 | *3%* | 1,330 | *2%* | 1,702 | *3%* | 6,813 | *3%* |
| Start-up (start-up and other costs) | 6,719 | *16%* | 11,499 | *20%* | 6,558 | *13%* | 33,270 | *14%* |
| Implementation | 34,512 | *81%* | 46,105 | *78%* | 42,588 | *84%* | 199,997 | *83%* |
| **COST CATEGORIES** |  |  |  |  |  |  |  |  |
| **Start-up** |  |  |  |  |  |  |  |  |
| S1. Trainings | 1,170 | 3% | 1,253 | 2% | 1,601 | 3% | 6,243 | 3% |
| S2. Sensitisation | 3,879 | 9% | 8,462 | 14% | 2,680 | 5% | 17,865 | 7% |
| *Total Start-up* | *5,049* | *12%* | *9,716* | *16%* | *4,280* | *8%* | *24,108* | *10%* |
| **Capital** |  |  |  |  |  |  |  |  |
| C1. Buildings and storage | 1,131 | 3% | 1,311 | 2% | 1,561 | 3% | 6,002 | 3% |
| C2. Equipment | 34 | 0% | 38 | 0% | 47 | 0% | 179 | 0% |
| C3. Vehicles | 9 | 0% | 9 | 0% | 20 | 0% | 36 | 0% |
| C4. Other capital costs | 0 | 0% | 0 | 0% | 0 | 0% | 0 | 0% |
| *Total Capital* | *1,174* | *3%* | *1,359* | *2%* | *1,627* | *3%* | *6,218* | *3%* |
| **Recurrent** |  |  |  |  |  |  |  |  |
| R1. Personnel – Headquarters IPO coordination | 8,440 | 20% | 9,724 | 16% | 11,547 | 23% | 45,035 | 19% |
| R2. Personnel – Headquarters IPO country | 11,845 | 28% | 13,748 | 23% | 16,207 | 32% | 63,206 | 26% |
| R3. Personnel – Headquarters IP | 2,897 | 7% | 3,386 | 6% | 3,963 | 8% | 15,456 | 6% |
| R4. Personnel – Field - HIVST distributors | 6,073 | 14% | 7,099 | 12% | 1,643 | 3% | 6,409 | 3% |
| R5. HIV self-testing kits | 2,193 | 5% | 8,444 | 14% | 5,033 | 10% | 49,840 | 21% |
| R6. Vehicle operation and maintenance | 1,606 | 4% | 1,739 | 3% | 1,541 | 3% | 15,257 | 6% |
| R7. Building operation and maintenance | 1,264 | 3% | 1,469 | 2% | 2,335 | 5% | 4,280 | 2% |
| R8. Other recurrent costs | 1,935 | 5% | 2,252 | 4% | 2,671 | 5% | 10,271 | 4% |
| *Total Recurrent* | *36,253* | *85%* | *47,859* | *81%* | *44,940* | *88%* | *209,754* | *87%* |
| **TOTAL ANNUAL COSTS** | **42,476** |  | **58,933** |  | **50,848** |  | **240,080** |  |
| HIVST kits distributed | 715 |  | 2,753 |  | 1,641 |  | 16,250 |  |
| **Average cost per HIVST kit distributed** | **59** |  | **21** |  | **31** |  | **15** |  |

CSO: Civil Society Organisation, IPO: International Partner Organisation, IP: Implementing Partner, HIVST: HIV Self-Testing kit, FSW: Female Sex workers, MSM: Men who have Sex with Men, PWUD: People who use drugs

**Appendix Table 2.c.** Observed total and average intervention costs by CSO and key groups – Mali (2/2)

|  | **CSO3** | | **CSO4** | | **CSO5** | | | |
| --- | --- | --- | --- | --- | --- | --- | --- | --- |
|  | **FSW** | | **MSM** | | **MSM** | | **FSW** | |
|  | **$** | **%** | **$** | **%** | **$** | **%** | **$** | **%** |
| **INTERVENTION PHASES** |  |  |  |  |  |  |  |  |
| Development | 2,833 | *3%* | 1,732 | *3%* | 754 | *3%* | 568 | *2%* |
| Start-up (start-up and other costs) | 24,581 | *22%* | 13,024 | *20%* | 3,331 | *11%* | 4,995 | *19%* |
| Implementation | 85,714 | *76%* | 50,009 | *77%* | 25,985 | *86%* | 20,849 | *79%* |
| **COST CATEGORIES** |  |  |  |  |  |  |  |  |
| **Start-up** |  |  |  |  |  |  |  |  |
| S1. Trainings | 2,481 | 2% | 1,521 | 2% | 560 | 2% | 560 | 2% |
| S2. Sensitisation | 18,285 | 16% | 9,171 | 14% | 1,706 | 6% | 3,687 | 14% |
| *Total Start-up* | *20,766* | *18%* | *10,692* | *17%* | *2,266* | *8%* | *4,247* | *16%* |
| **Capital** |  |  |  |  |  |  |  |  |
| C1. Buildings and storage | 2,399 | 2% | 1,591 | 2% | 646 | 2% | 512 | 2% |
| C2. Equipment | 72 | 0% | 46 | 0% | 19 | 0% | 15 | 0% |
| C3. Vehicles | 48 | 0% | 29 | 0% | 6 | 0% | 6 | 0% |
| C4. Other capital costs | 0 | 0% | 0 | 0% | 0 | 0% | 0 | 0% |
| *Total Capital* | *2,518* | *2%* | *1,666* | *3%* | *672* | *2%* | *534* | *2%* |
| **Recurrent** |  |  |  |  |  |  |  |  |
| R1. Personnel – Headquarters IPO coordination | 17,899 | 16% | 11,797 | 18% | 6,153 | 20% | 3,441 | 13% |
| R2. Personnel – Headquarters IPO country | 25,120 | 22% | 16,678 | 26% | 5,672 | 19% | 5,672 | 21% |
| R3. Personnel – Headquarters IP | 6,143 | 5% | 4,107 | 6% | 1,387 | 5% | 1,387 | 5% |
| R4. Personnel – Field - HIVST distributors | 2,547 | 2% | 1,703 | 3% | 5,528 | 18% | 5,528 | 21% |
| R5. HIV self-testing kits | 23,092 | 20% | 8,244 | 13% | 5,462 | 18% | 3,055 | 12% |
| R6. Vehicle operation and maintenance | 8,258 | 7% | 5,365 | 8% | 1,101 | 4% | 1,101 | 4% |
| R7. Building operation and maintenance | 2,681 | 2% | 1,782 | 3% | 722 | 2% | 572 | 2% |
| R8. Other recurrent costs | 4,104 | 4% | 2,732 | 4% | 1,106 | 4% | 876 | 3% |
| *Total Recurrent* | *89,844* | *79%* | *52,408* | *81%* | *27,132* | *90%* | *21,632* | *82%* |
| **TOTAL ANNUAL COSTS** | **113,128** |  | **64,765** |  | **30,070** |  | **26,413** |  |
| HIVST kits distributed | 7,529 |  | 2,688 |  | 1,781 |  | 996 |  |
| **Average cost per HIVST kit distributed** | **15** |  | **24** |  | **17** |  | **27** |  |

CSO: Civil Society Organisation, IPO: International Partner Organisation, IP: Implementing Partner, HIVST: HIV Self-Testing kit, FSW: Female Sex workers, MSM: Men who have Sex with Men, PWUD: People who use drugs

**Appendix Figure 1.a.** Tornado diagrams of findings from deterministic sensitivity analysis in Côte d’Ivoire


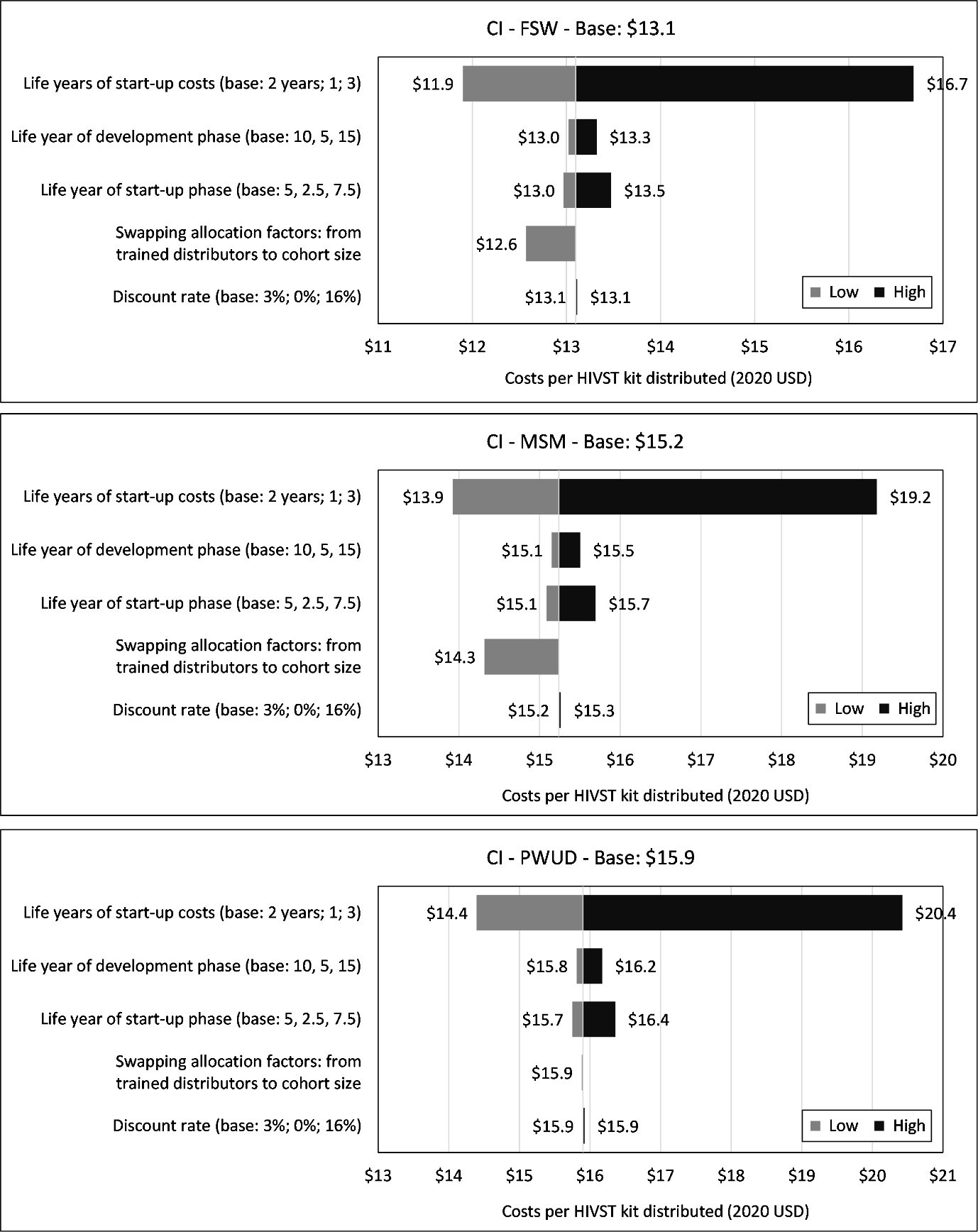


HIVST: HIV Self-Testing kit, FSW: Female Sex workers, MSM: Men who have Sex with Men, PWUD: People who use drugs

**Appendix Figure 1.b.** Tornado diagrams of findings from deterministic sensitivity analysis in Senegal


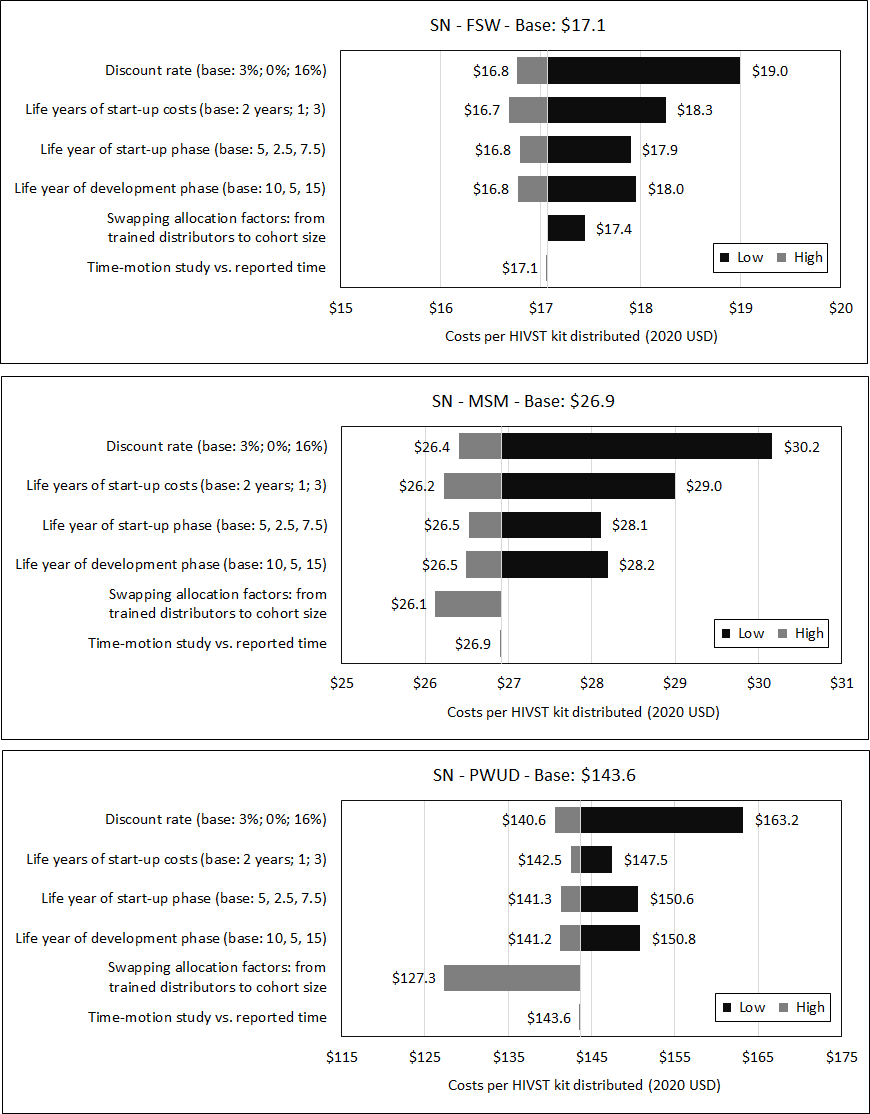


HIVST: HIV Self-Testing kit, FSW: Female Sex workers, MSM: Men who have Sex with Men, PWUD: People who use drugs

**Appendix Figure 1.c.** Tornado diagrams of findings from deterministic sensitivity analysis in Mali


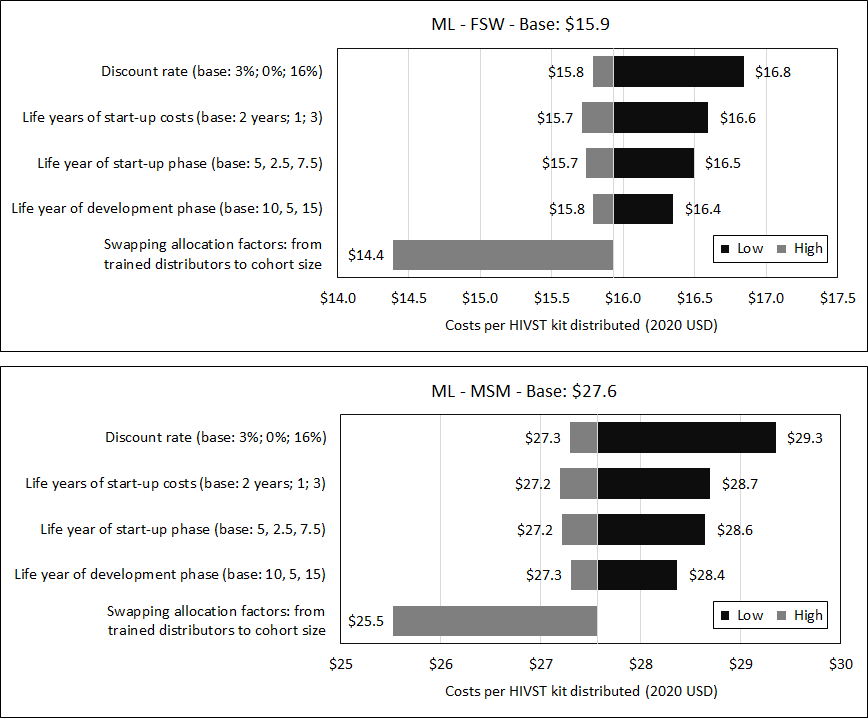


HIVST: HIV Self-Testing kit, FSW: Female Sex workers, MSM: Men who have Sex with Men, PWUD: People who use drugs

**Appendix Table 3.a.** Total and average intervention costs in transition and at scale-up by key group and scale-up year – Côte d’Ivoire

|  | **Côte d’Ivoire** | | | | | |
| --- | --- | --- | --- | --- | --- | --- |
|  | **2021** | | | | | |
|  | **FSW** | | **MSM** | | **PWUD** | |
| **Intervention level and costs** | **$** | **%** | **$** | **%** | **$** | **%** |
| International level - Fixed costs (S2, R1) | 62,455 | 8% | 27,766 | 7% | 8,946 | 5% |
| National level - Fixed costs (C1-C4, S2, R2) | 88,073 | 11% | 39,347 | 10% | 12,574 | 7% |
| National level - Variable costs (S1) | 111,930 | 14% | 46,063 | 12% | 17,407 | 10% |
| National level - Variable costs (R6-R8) | 17,712 | 2% | 9,720 | 3% | 15,232 | 9% |
| Sub-national - Implementing partners (R3) | 307,960 | 37% | 144,216 | 38% | 73,789 | 44% |
| Local - HIVST distribution areas (R4) | 23,620 | 3% | 12,130 | 3% | 11,462 | 7% |
| Local - HIVST distribution areas (R5) | 213,489 | 26% | 98,398 | 26% | 29,419 | 17% |
| **Total costs** | 825,239 |  | 377,641 |  | 168,828 |  |
| **Scale** | 81,174 |  | 37,414 |  | 11,186 |  |
| **Average costs** | 10 |  | 10 |  | 15 |  |
|  | **2022** | | | | | |
|  | **FSW** | | **MSM** | | **PWUD** | |
| **Intervention level and costs** | **$** | **%** | **$** | **%** | **$** | **%** |
| International level - Fixed costs (S2, R1) | 62,455 | 6% | 27,766 | 6% | 8,946 | 4% |
| National level - Fixed costs (C1-C4, S2, R2) | 88,073 | 8% | 39,347 | 8% | 12,574 | 5% |
| National level - Variable costs (S1) | 74,717 | 7% | 28,690 | 6% | 12,528 | 5% |
| National level - Variable costs (R6-R8) | 26,213 | 2% | 14,008 | 3% | 23,273 | 10% |
| Sub-national - Implementing partners (R3) | 455,768 | 43% | 207,836 | 44% | 112,741 | 48% |
| Local - HIVST distribution areas (R4) | 34,956 | 3% | 17,481 | 4% | 17,512 | 8% |
| Local - HIVST distribution areas (R5) | 315,954 | 30% | 141,806 | 30% | 44,949 | 19% |
| **Total costs** | 1,058,137 |  | 476,935 |  | 232,523 |  |
| **Scale** | 120,135 |  | 53,919 |  | 17,091 |  |
| **Average costs** | 9 |  | 9 |  | 14 |  |
|  | **2023** | | | | | |
|  | **FSW** | | **MSM** | | **PWUD** | |
| **Intervention level and costs** | **$** | **%** | **$** | **%** | **$** | **%** |
| International level - Fixed costs (S2, R1) | 62,455 | 4% | 27,766 | 4% | 8,946 | 3% |
| National level - Fixed costs (C1-C4, S2, R2) | 88,073 | 6% | 39,347 | 6% | 12,574 | 4% |
| National level - Variable costs (S1) | 100,175 | 7% | 52,436 | 7% | 16,958 | 5% |
| National level - Variable costs (R6-R8) | 37,611 | 3% | 21,845 | 3% | 34,157 | 10% |
| Sub-national - Implementing partners (R3) | 653,937 | 45% | 324,112 | 45% | 165,468 | 50% |
| Local - HIVST distribution areas (R4) | 50,155 | 3% | 27,262 | 4% | 25,702 | 8% |
| Local - HIVST distribution areas (R5) | 453,332 | 31% | 221,140 | 31% | 65,970 | 20% |
| **Total costs** | 1,445,738 |  | 713,908 |  | 329,775 |  |
| **Scale** | 172,370 |  | 84,084 |  | 25,084 |  |
| **Average costs** | 8 |  | 8 |  | 13 |  |

S1: Trainings, S2: Sensitisation, C1: Buildings and storage, C2: Equipment, C3: Vehicles, C4: Other capital costs, R1: Personnel & Per diems – Headquarters International Partner Organisation (IPO) coordination, R2: Personnel & Per diems – Headquarters IPO country, R3: Personnel & Per diems – Headquarters Implementing partner, R4: Personnel & Per diems – Field - HIVST distributors, R5: HIV self-testing kits, R6: Vehicle operation and maintenance, R7: Building operation and maintenance, R8: Other recurrent costs

HIVST: HIV Self-Testing kit, FSW: Female Sex workers, MSM: Men who have Sex with Men, PWUD: People who use drugs

**Appendix Table 3.b.** Total and average intervention costs in transition and at scale-up by key group and scale-up year – Senegal

|  | **Senegal** | | | | | |
| --- | --- | --- | --- | --- | --- | --- |
|  | **2021** | | | | | |
|  | **FSW** | | **MSM** | | **PWUD** | |
| **Intervention level and costs** | **$** | **%** | **$** | **%** | **$** | **%** |
| International level - Fixed costs (S2, R1) | 32,639 | 11% | 23,681 | 6% | 18,043 | 9% |
| National level - Fixed costs (C1-C4, S2, R2) | 41,676 | 15% | 32,696 | 8% | 35,547 | 19% |
| National level - Variable costs (S1) | 9,092 | 3% | 26,630 | 7% | 6,302 | 3% |
| National level - Variable costs (R6-R8) | 35,020 | 12% | 67,433 | 17% | 27,543 | 14% |
| Sub-national - Implementing partners (R3) | 61,964 | 22% | 122,533 | 31% | 64,128 | 33% |
| Local - HIVST distribution areas (R4) | 37,900 | 13% | 74,946 | 19% | 29,174 | 15% |
| Local - HIVST distribution areas (R5) | 65,761 | 23% | 50,592 | 13% | 11,165 | 6% |
| **Total costs** | 284,051 |  | 398,511 |  | 191,902 |  |
| **Scale** | 21,351 |  | 16,426 |  | 3,625 |  |
| **Average costs** | 13 |  | 24 |  | 53 |  |
|  | **2022** | | | | | |
|  | **FSW** | | **MSM** | | **PWUD** | |
| **Intervention level and costs** | **$** | **%** | **$** | **%** | **$** | **%** |
| International level - Fixed costs (S2, R1) | 32,639 | 11% | 23,681 | 6% | 18,043 | 9% |
| National level - Fixed costs (C1-C4, S2, R2) | 41,676 | 14% | 32,696 | 9% | 35,547 | 17% |
| National level - Variable costs (S1) | 1,026 | 0% | 796 | 0% | 1,229 | 1% |
| National level - Variable costs (R6-R8) | 37,482 | 13% | 69,108 | 18% | 31,282 | 15% |
| Sub-national - Implementing partners (R3) | 66,320 | 23% | 125,577 | 33% | 72,832 | 36% |
| Local - HIVST distribution areas (R4) | 40,564 | 14% | 76,808 | 20% | 33,134 | 16% |
| Local - HIVST distribution areas (R5) | 70,384 | 24% | 51,849 | 14% | 12,680 | 6% |
| **Total costs** | 290,091 |  | 380,514 |  | 204,746 |  |
| **Scale** | 22,852 |  | 16,834 |  | 4,117 |  |
| **Average costs** | 13 |  | 23 |  | 50 |  |
|  | **2023** | | | | | |
|  | **FSW** | | **MSM** | | **PWUD** | |
| **Intervention level and costs** | **$** | **%** | **$** | **%** | **$** | **%** |
| International level - Fixed costs (S2, R1) | 32,639 | 11% | 23,681 | 5% | 18,043 | 8% |
| National level - Fixed costs (C1-C4, S2, R2) | 41,676 | 14% | 32,696 | 7% | 35,547 | 16% |
| National level - Variable costs (S1) | 988 | 0% | 7,612 | 2% | 1,299 | 1% |
| National level - Variable costs (R6-R8) | 39,852 | 13% | 85,122 | 18% | 35,233 | 16% |
| Sub-national - Implementing partners (R3) | 70,514 | 23% | 154,677 | 33% | 82,031 | 37% |
| Local - HIVST distribution areas (R4) | 43,129 | 14% | 94,607 | 20% | 37,319 | 17% |
| Local - HIVST distribution areas (R5) | 74,835 | 25% | 63,864 | 14% | 14,282 | 6% |
| **Total costs** | 303,632 |  | 462,259 |  | 223,752 |  |
| **Scale** | 24,297 |  | 20,735 |  | 4,637 |  |
| **Average costs** | 12 |  | 22 |  | 48 |  |

S1: Trainings, S2: Sensitisation, C1: Buildings and storage, C2: Equipment, C3: Vehicles, C4: Other capital costs, R1: Personnel & Per diems – Headquarters International Partner Organisation (IPO) coordination, R2: Personnel & Per diems – Headquarters IPO country, R3: Personnel & Per diems – Headquarters Implementing partner, R4: Personnel & Per diems – Field - HIVST distributors, R5: HIV self-testing kits, R6: Vehicle operation and maintenance, R7: Building operation and maintenance, R8: Other recurrent costs

HIVST: HIV Self-Testing kit, FSW: Female Sex workers, MSM: Men who have Sex with Men, PWUD: People who use drugs

**Appendix Table 3.c.** Total and average intervention costs in transition and at scale-up by key group and scale-up year – Mali

|  | **Mali** | | | |
| --- | --- | --- | --- | --- |
|  | **2021** | | | |
|  | **FSW** | | **MSM** | |
| **Intervention level and costs** | **$** | **%** | **$** | **%** |
| International level - Fixed costs (S2, R1) | 116,572 | 12% | 52,548 | 13% |
| National level - Fixed costs (C1-C4, S2, R2) | 126,200 | 13% | 58,366 | 14% |
| National level - Variable costs (S1) | 34,350 | 3% | 15,537 | 4% |
| National level - Variable costs (R6-R8) | 225,175 | 23% | 101,531 | 25% |
| Sub-national - Implementing partners (R3) | 112,339 | 11% | 51,918 | 13% |
| Local - HIVST distribution areas (R4) | 91,938 | 9% | 62,817 | 15% |
| Local - HIVST distribution areas (R5) | 288,941 | 29% | 70,674 | 17% |
| **Total costs** | 995,515 |  | 413,392 |  |
| **Scale** | 93,812 |  | 22,946 |  |
| **Average costs** | 11 |  | 18 |  |
|  | **2022** | | | |
|  | **FSW** | | **MSM** | |
| **Intervention level and costs** | **$** | **%** | **$** | **%** |
| International level - Fixed costs (S2, R1) | 116,572 | 10% | 52,548 | 11% |
| National level - Fixed costs (C1-C4, S2, R2) | 126,200 | 11% | 58,366 | 12% |
| National level - Variable costs (S1) | 10,917 | 1% | 4,959 | 1% |
| National level - Variable costs (R6-R8) | 279,940 | 24% | 126,226 | 27% |
| Sub-national - Implementing partners (R3) | 139,661 | 12% | 64,546 | 14% |
| Local - HIVST distribution areas (R4) | 114,298 | 10% | 78,096 | 17% |
| Local - HIVST distribution areas (R5) | 359,214 | 31% | 87,863 | 19% |
| **Total costs** | 1,146,802 |  | 472,604 |  |
| **Scale** | 116,628 |  | 28,527 |  |
| **Average costs** | 10 |  | 17 |  |
|  | **2023** | | | |
|  | **FSW** | | **MSM** | |
| **Intervention level and costs** | **$** | **%** | **$** | **%** |
| International level - Fixed costs (S2, R1) | 116,572 | 9% | 52,548 | 10% |
| National level - Fixed costs (C1-C4, S2, R2) | 126,200 | 10% | 58,366 | 12% |
| National level - Variable costs (S1) | 5,622 | 0% | 2,554 | 1% |
| National level - Variable costs (R6-R8) | 308,143 | 25% | 138,943 | 27% |
| Sub-national - Implementing partners (R3) | 153,731 | 12% | 71,049 | 14% |
| Local - HIVST distribution areas (R4) | 125,814 | 10% | 85,964 | 17% |
| Local - HIVST distribution areas (R5) | 395,404 | 32% | 96,715 | 19% |
| **Total costs** | 1,231,486 |  | 506,138 |  |
| **Scale** | 128,378 |  | 31,401 |  |
| **Average costs** | 10 |  | 16 |  |

S1: Trainings, S2: Sensitisation, C1: Buildings and storage, C2: Equipment, C3: Vehicles, C4: Other capital costs, R1: Personnel & Per diems – Headquarters International Partner Organisation (IPO) coordination, R2: Personnel & Per diems – Headquarters IPO country, R3: Personnel & Per diems – Headquarters Implementing partner, R4: Personnel & Per diems – Field - HIVST distributors, R5: HIV self-testing kits, R6: Vehicle operation and maintenance, R7: Building operation and maintenance, R8: Other recurrent costs

HIVST: HIV Self-Testing kit, FSW: Female Sex workers, MSM: Men who have Sex with Men, PWUD: People who use drugs

**Appendix Figure 2.a.** Average cost at scale per HIVST kit distributed by key group and scale-up year - Scenario analysis in Côte d’Ivoire

*Scenario 1*: We anticipate that programmatic objectives might not be reached. Accounting for this would provide more nuanced scale economies, and we applied different percentages for reaching targets

*Scenario 2*: International Partner Organisation’s goal to progressively disengage to promote local programme ownership overtime was considered. Note that we still account for 15% of international costs in 2023 because we assume another coordination component will still exist (and incur costs) within the local health system at central level. Year 2023 would then represent what it costs for the country to support HIVST post-ATLAS

*Scenario 3*: We assessed the impact of optimising delivery channels by simplifying the model of partners/sub-partners and decreased civil society organisation’s headquarter costs by 20%, which is reasonable to assume when evaluating interventions transitioning from pilot (ATLAS) to routine implementation phase

*Scenario 4*: We conducted country-specific simulations to account for varying HIVST kit cost for each year considering factors such as bulk buying, maritime provision instead of airways (except Mali), and integrating HIVST delivery chain with other health supplies

*All*: We combined all scenarios (1 to 4) to assess the global impact on average costs at scale per key population and scale-up year

*Baseline scenario*: All parameters above are unchanged (100% of their original value)


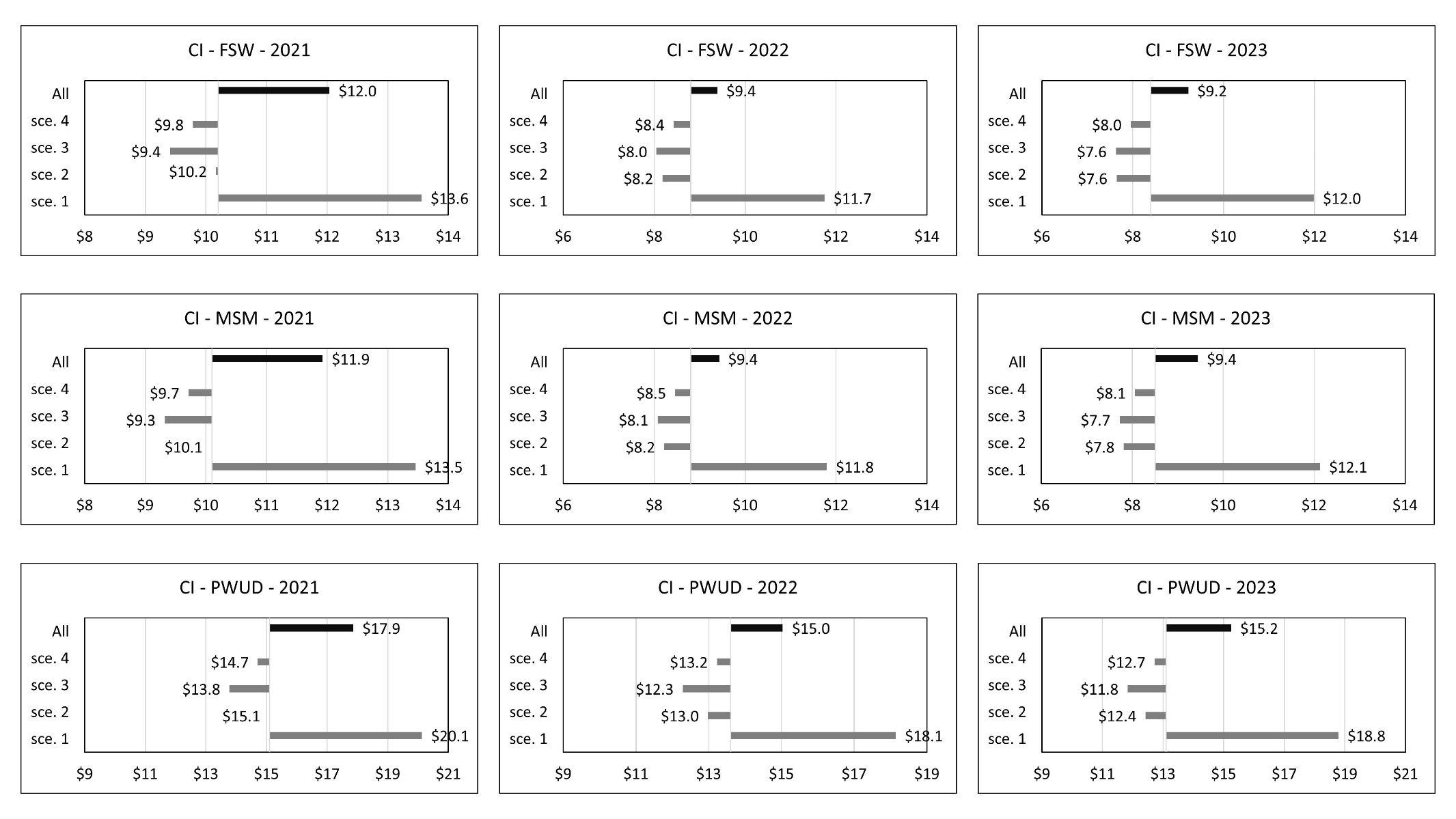


CI: Côte d’Ivoire, SN: Senegal, ML: Mali, HIVST: HIV Self-Testing kit, FSW: Female Sex workers, MSM: Men who have Sex with Men, PWUD: People who use drugs

**Appendix Figure 2.b.** Average cost at scale per HIVST kit distributed by key group and scale-up year - Scenario analysis in Senegal


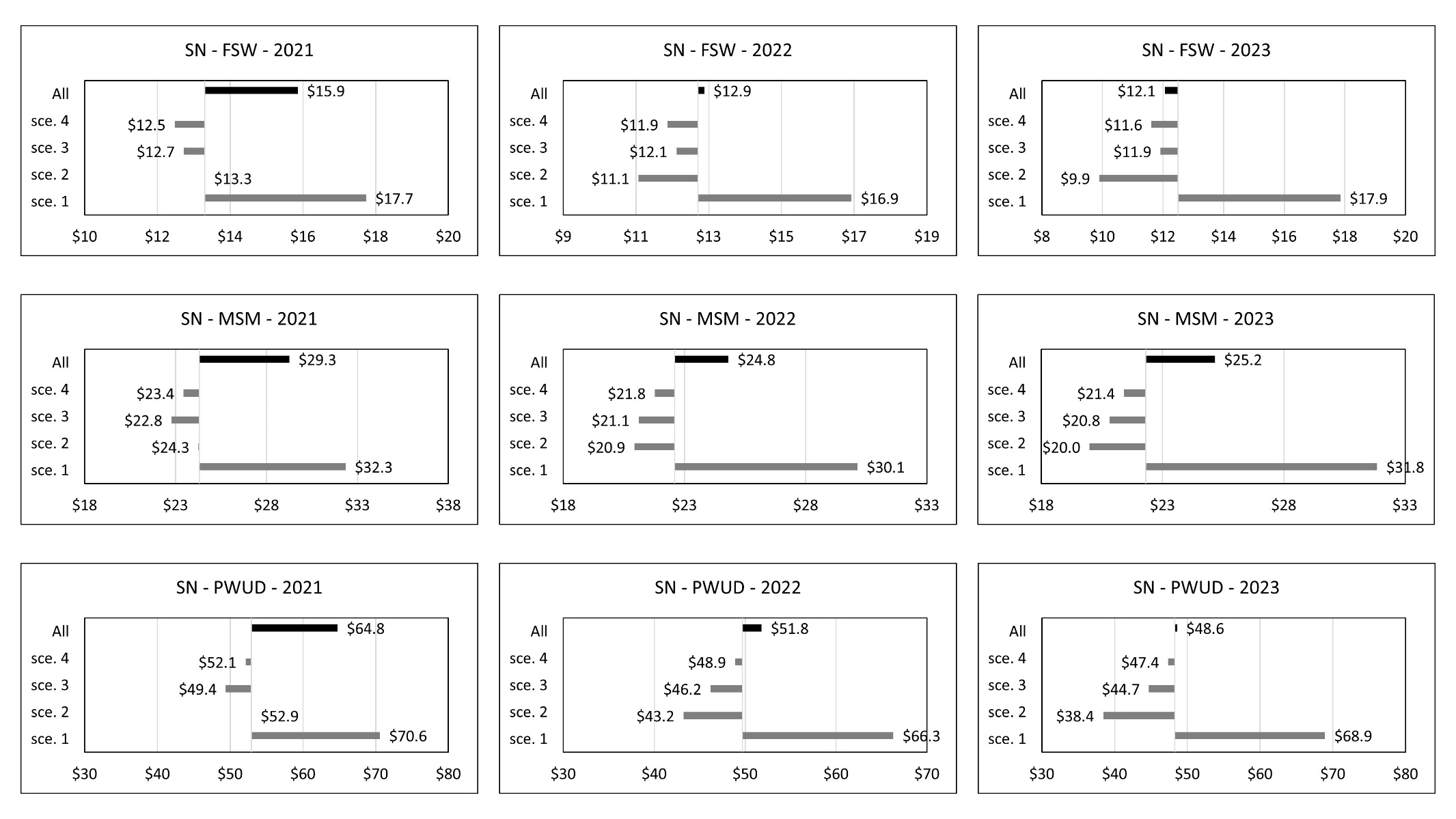
CI: Côte d’Ivoire, SN: Senegal, ML: Mali, HIVST: HIV Self-Testing kit, FSW: Female Sex workers, MSM: Men who have Sex with Men, PWUD: People who use drugs

**Appendix Figure 2.c.** Average cost at scale per HIVST kit distributed by key group and scale-up year - Scenario analysis in Mali


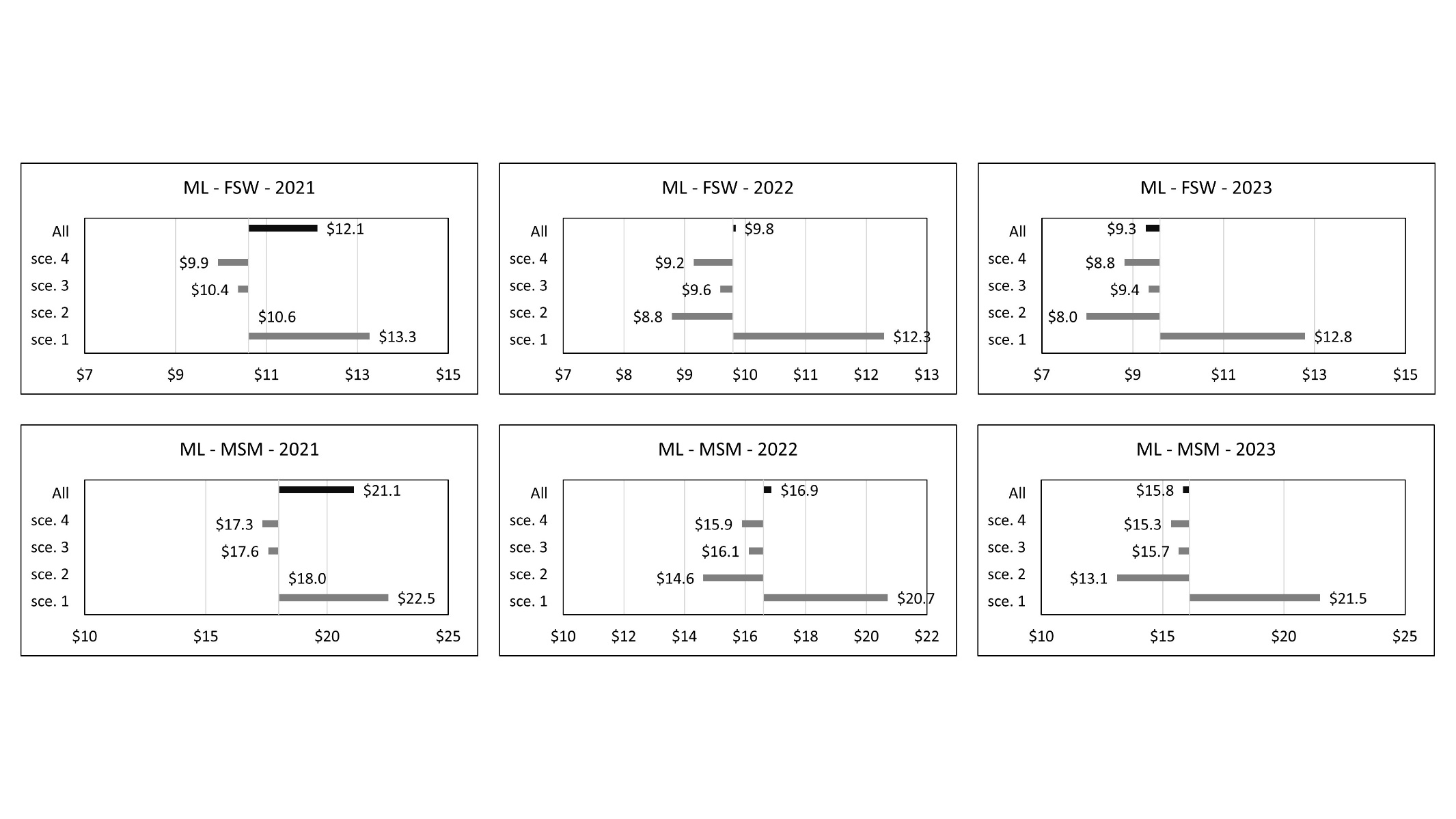


CI: Côte d’Ivoire, SN: Senegal, ML: Mali, HIVST: HIV Self-Testing kit, FSW: Female Sex workers, MSM: Men who have Sex with Men, PWUD: People who use drugs
